# Supplementary material for: The Queensland Twin Adolescent Brain Project, a longitudinal study of adolescent brain development
Source: Sci Data. 2023 Apr 8;10:195. doi: 10.1038/s41597-023-02038-w (PMC10082846; doi:10.1038/s41597-023-02038-w)
Supplement: Supplementary file 1 — Supplementary Materials [file 41597_2023_2038_MOESM1_ESM.docx]

**Supplementary Material**

*The Queensland Twin Adolescent Brain Project, a longitudinal study of adolescent brain development*

Table of Contents

[**Supplementary Table 1** Overview of the QTAB OpenNeuro dataset (imaging). 4](#_Toc127890992)

[**Supplementary Table 2** Overview of the QTAB Zenodo dataset (non-imaging phenotypes). 5](#_Toc127890993)

[**Supplementary Table 3** Overview of questionnaire scale items. 6](#_Toc127890994)

[Pubertal Developmental Scale (PDS) 6](#_Toc127890995)

[Sexual Maturation Scale (SMS) 6](#_Toc127890996)

[Prospective and Retrospective Memory Questionnaire for Children (PRMQC) 6](#_Toc127890997)

[Empathy Questionnaire for Children and Adolescents (EmQue-CA) 7](#_Toc127890998)

[Spence Children’s Anxiety Scale (SCAS) 7](#_Toc127890999)

[Short Moods and Feelings Questionnaire (SMFQ) 9](#_Toc127891000)

[Somatic and Psychological Health Report (SPHERE-21) 9](#_Toc127891001)

[Short UPPS-P Impulsive Behaviours Scale in Children (IBS) 10](#_Toc127891002)

[Strength and Difficulties Questionnaire (SDQ) 10](#_Toc127891003)

[Australian-adapted Hierarchical Personality Inventory for Children (HiPIC-A) 11](#_Toc127891004)

[Autism Spectrum Quotient – 10 items (AQ-10) (Adolescent) 12](#_Toc127891005)

[Children’s Response Styles Questionnaire – rumination subscale (CRSQ) 12](#_Toc127891006)

[Children’s Attributional Style Questionnaire – Revised (CASQ-R) 12](#_Toc127891007)

[Early Adolescent Temperament Questionnaire – Revised (EATQ-R) 13](#_Toc127891008)

[Multidimensional Scale of Perceived Social Support (MSPSS) 14](#_Toc127891009)

[Alabama Parenting Questionnaire (APQ) 15](#_Toc127891010)

[McMaster Family Assessment Device (FAD) 16](#_Toc127891011)

[Daily Life Stressors Scale (DLSS) 18](#_Toc127891012)

[Gatehouse Bullying Scale (GBS) 19](#_Toc127891013)

[Childhood Life Events Questionnaire (CLEQ) 19](#_Toc127891014)

[Prenatal Stress Exposure Scale (PSES) 19](#_Toc127891015)

[Parental Stress Scale (PaSS) 21](#_Toc127891016)

[List of Threatening Experiences (LTE) 21](#_Toc127891017)

[Pediatric Daytime Sleepiness Scale (PDSS) 21](#_Toc127891018)

[Sleep Disturbances Scale for Children (SDSC) 22](#_Toc127891019)

[Sleep behaviours across early childhood 22](#_Toc127891020)

[Active and Passive Social Media Use (APSMU) 23](#_Toc127891021)

[UCLA Brief COVID-19 Screen for Child/Adolescent PTSD 23](#_Toc127891022)

[Perceived Stress Scale (PSS) 23](#_Toc127891023)

[Brief Resilience Scale (BRS) 24](#_Toc127891024)

| Filename | Description |
| --- | --- |
| qtab |  |
| ├──.bidsignore | Files ignored by the BIDS validator |
| ├── CHANGES | Change log |
| ├── README | README file |
| ├── dataset_description.json | Brief dataset description |
| ├── participants.json | Data dictionary for participants.tsv |
| ├── participants.tsv | Participant demographics |
| ├── derivitives |  |
| │   ├── UNIT1_denoised |  |
| │   │   └── sub-0001 |  |
| │   │      ├── ses-01 |  |
| │   │      │ └──anat |  |
| │   │      │ └── sub-0001_ses-01_UNIT1_unbiased_clean.nii.gz | MP2RAGE denoised uniform scan (AFNI implementation) |
| │   │      └── ses-02 |  |
| │   │      └──anat |  |
| │   │      └── sub-0001_ses-02_UNIT1_unbiased_clean.nii.gz | MP2RAGE denoised uniform scan (AFNI implementation) |
| │   ├── mriqc |  |
| │   │   ├── ses-01_task-rest_bold.json | Data dictionary for ses-01_task-rest_bold.tsv |
| │   │   ├── ses-01_task-rest_bold.tsv | mriqc image quality metrics for session 1 rest scans |
| │   │   ├── ses-02_task-emotionalconflict_bold.json | Data dictionary for ses-01_task-emotionalconflict_bold.json |
| │   │   ├── ses-02_task-emotionalconflict_bold.tsv | mriqc image quality metrics for session 2 emotional conflict task scan |
| │   │   ├── ses-02_task-partlycloudy_bold.json | Data dictionary for ses-02_task-partlycloudy_bold.tsv |
| │   │   ├── ses-02_task-partlycloudy_bold.tsv | mriqc image quality metrics for session 2 partly cloudy task scan |
| │   │   ├── ses-02_task-rest_bold.json | Data dictionary for ses-02_task-rest_bold.tsv |
| │   │   └── ses-02_task-rest_bold.tsv | mriqc image quality metrics for session 2 rest scans |
| │   ├── mrtix3 |  |
| │   │   ├── ses-01_dwi.json | Data dictionary for ses-01_dwi.tsv |
| │   │   ├── ses-01_dwi.tsv | EDDY QC measures for session 1 diffusion scans |
| │   │   ├── ses-02_dwi.json | Data dictionary for ses-02_dwi.tsv |
| │   │   └── ses-02_dwi.tsv | EDDY QC measures for session 2 diffusion scans |
| │   └── visual_qc |  |
| │   ├── anat_ses-01_qc.json | Data dictionary for anat_ses-01_qc.json |
| │   ├── anat_ses-01_qc.tsv | Visual quality ratings for session 1 anatomical scans |
| │   ├── anat_ses-02_qc.json | Data dictionary for anat_ses-02_qc.json |
| │   └── anat_ses-02_qc.tsv | Visual quality ratings for session 2 anatomical scans |
| └── sub-0001 |  |
| ├── ses-01 |  |
| │   ├── anat |  |
| │   │   ├── sub-0001_ses-01_FLAIR.json | FLAIR acquisition parameters |
| │   │   ├── sub-0001_ses-01_FLAIR.nii.gz | FLAIR scan |
| │   │   ├── sub-0001_ses-01_T2w.json | T2w acquisition parameters |
| │   │   ├── sub-0001_ses-01_T2w.nii.gz | T2w scan |
| │   │   ├── sub-0001_ses-01_T2w_TSE_run-01.json | TSE acquisition parameters |
| │   │   ├── sub-0001_ses-01_T2w_TSE_run-01.nii.gz | TSE scan |
| │   │   ├── sub-0001_ses-01_T2w_TSE_run-02.json | TSE acquisition parameters |
| │   │   ├── sub-0001_ses-01_T2w_TSE_run-02.nii.gz | TSE scan |
| │   │   ├── sub-0001_ses-01_T2w_TSE_run-03.json | TSE acquisition parameters |
| │   │   ├── sub-0001_ses-01_T2w_TSE_run-03.nii.gz | TSE scan |
| │   │   ├── sub-0001_ses-01_UNIT1.json | MP2RAGE uniform acquisition parameters |
| │   │   ├── sub-0001_ses-01_UNIT1.nii.gz | MP2RAGE uniform scan |
| │   │   ├── sub-0001_ses-01_UNIT1_denoised.json | MP2RAGE denoised uniform acquisition parameters |
| │   │   ├── sub-0001_ses-01_UNIT1_denoised.nii.gz | MP2RAGE denoised uniform scan |
| │   │   ├── sub-0001_ses-01_inv-1_MP2RAGE.json | MP2RAGE inversion time 1 acquisition parameters |
| │   │   ├── sub-0001_ses-01_inv-1_MP2RAGE.nii.gz | MP2RAGE inversion time 1 scan |
| │   │   ├── sub-0001_ses-01_inv-2_MP2RAGE.json | MP2RAGE inversion time 2 acquisition parameters |
| │   │   ├── sub-0001_ses-01_inv-2_MP2RAGE.nii.gz | MP2RAGE inversion time 2 scan |
| │   │   └── sub-0001_ses-01_inv-2_MP2RAGE_defacemask.nii.gz | Mask used to deface anatomical scans (based on inv-2_MP2RAGE) |
| │   ├── dwi |  |
| │   │   ├── sub-0001_ses-01_dir-AP_run-01_dwi.bval | Diffusion b-value (AP direction, first acquisition) |
| │   │   ├── sub-0001_ses-01_dir-AP_run-01_dwi.bvec | Diffusion b-vector (AP direction, first acquisition) |
| │   │   ├── sub-0001_ses-01_dir-AP_run-01_dwi.json | Diffusion acquisition parameters (AP direction, first acquisition) |
| │   │   ├── sub-0001_ses-01_dir-AP_run-01_dwi.nii.gz | Diffusion scan (AP direction, first acquisition) |
| │   │   ├── sub-0001_ses-01_dir-AP_run-02_dwi.bval | Diffusion b-value (AP direction, second acquisition) |
| │   │   ├── sub-0001_ses-01_dir-AP_run-02_dwi.bvec | Diffusion b-vector (AP direction, second acquisition) |
| │   │   ├── sub-0001_ses-01_dir-AP_run-02_dwi.json | Diffusion acquisition parameters (AP direction, second acquisition) |
| │   │   ├── sub-0001_ses-01_dir-AP_run-02_dwi.nii.gz | Diffusion scan (AP direction, second acquisition) |
| │   │   ├── sub-0001_ses-01_dir-PA_run-01_dwi.bval | Diffusion b-value (PA direction, first acquisition) |
| │   │   ├── sub-0001_ses-01_dir-PA_run-01_dwi.bvec | Diffusion b-vector (PA direction, first acquisition) |
| │   │   ├── sub-0001_ses-01_dir-PA_run-01_dwi.json | Diffusion acquisition parameters (PA direction, first acquisition) |
| │   │   ├── sub-0001_ses-01_dir-PA_run-01_dwi.nii.gz | Diffusion scan (PA direction, first acquisition) |
| │   │   ├── sub-0001_ses-01_dir-PA_run-02_dwi.bval | Diffusion b-value (PA direction, second acquisition) |
| │   │   ├── sub-0001_ses-01_dir-PA_run-02_dwi.bvec | Diffusion b-vector (PA direction, second acquisition) |
| │   │   ├── sub-0001_ses-01_dir-PA_run-02_dwi.json | Diffusion acquisition parameters (PA direction, second acquisition) |
| │   │   └── sub-0001_ses-01_dir-PA_run-02_dwi.nii.gz | Diffusion scan (PA direction, second acquisition) |
| │   ├── func |  |
| │   │   ├── sub-0001_ses-01_task-rest_dir-AP_bold.json | rs-fMRI acquisition parameters (AP direction) |
| │   │   ├── sub-0001_ses-01_task-rest_dir-AP_bold.nii.gz | rs-fMRI scan (AP direction) |
| │   │   ├── sub-0001_ses-01_task-rest_dir-PA_bold.json | rs-fMRI acquisition parameters (PA direction) |
| │   │   └── sub-0001_ses-01_task-rest_dir-PA_bold.nii.gz | rs-fMRI scan (PA direction) |
| │   ├── perf |  |
| │   │   ├── sub-0001_ses-01_asl.json | ASL acquisition parameters |
| │   │   ├── sub-0001_ses-01_asl.nii.gz | ASL scan |
| │   │   ├── sub-0001_ses-01_aslcontext.tsv | Volume types of asl scan |
| │   │   ├── sub-0001_ses-01_m0scan.json | M0 acquisition parameters |
| │   │   └── sub-0001_ses-01_m0scan.nii.gz | M0 scan |
| │   └── swi |  |
| │   ├── sub-0001_ses-01_minIP.json | Minimum intensity projection acquisition parameters |
| │   ├── sub-0001_ses-01_minIP.nii.gz | Minimum intensity projection scan |
| │   ├── sub-0001_ses-01_part-mag_GRE.json | Magnitude acquisition parameters |
| │   ├── sub-0001_ses-01_part-mag_GRE.nii.gz | Magnitude scan |
| │   ├── sub-0001_ses-01_part-phase_GRE.json | Phase acquisition parameters |
| │   ├── sub-0001_ses-01_part-phase_GRE.nii.gz | Phase scan |
| │   ├── sub-0001_ses-01_swi.json | Combined magnitude and phase acquisition parameters |
| │   └── sub-0001_ses-01_swi.nii.gz | Combined magnitude and phase scan |
| └── ses-02 |  |
| ├── anat |  |
| │   ├── sub-0001_ses-02_FLAIR.json | FLAIR acquisition parameters |
| │   ├── sub-0001_ses-02_FLAIR.nii.gz | FLAIR scan |
| │   ├── sub-0001_ses-02_T2w.json | T2w acquisition parameters |
| │   ├── sub-0001_ses-02_T2w.nii.gz | T2w scan |
| │   ├── sub-0001_ses-02_T2w_TSE_run-01.json | TSE acquisition parameters |
| │   ├── sub-0001_ses-02_T2w_TSE_run-01.nii.gz | TSE scan |
| │   ├── sub-0001_ses-02_T2w_TSE_run-02.json | TSE acquisition parameters |
| │   ├── sub-0001_ses-02_T2w_TSE_run-02.nii.gz | TSE scan |
| │   ├── sub-0001_ses-02_UNIT1.json | MP2RAGE uniform acquisition parameters |
| │   ├── sub-0001_ses-02_UNIT1.nii.gz | MP2RAGE uniform scan |
| │   ├── sub-0001_ses-02_UNIT1_denoised.json | MP2RAGE denoised uniform acquisition parameters |
| │   ├── sub-0001_ses-02_UNIT1_denoised.nii.gz | MP2RAGE denoised uniform scan |
| │   ├── sub-0001_ses-02_inv-1_MP2RAGE.json | MP2RAGE inversion time 1 acquisition parameters |
| │   ├── sub-0001_ses-02_inv-1_MP2RAGE.nii.gz | MP2RAGE inversion time 1 scan |
| │   ├── sub-0001_ses-02_inv-2_MP2RAGE.json | MP2RAGE inversion time 2 acquisition parameters |
| │   ├── sub-0001_ses-02_inv-2_MP2RAGE.nii.gz | MP2RAGE inversion time 2 scan |
| │   └── sub-0001_ses-02_inv-2_MP2RAGE_defacemask.nii.gz | Mask used to deface anatomical scans (based on inv-2_MP2RAGE) |
| ├── dwi |  |
| │   ├── sub-0001_ses-02_dir-AP_run-01_dwi.bval | Diffusion b-value (AP direction, first acquisition) |
| │   ├── sub-0001_ses-02_dir-AP_run-01_dwi.bvec | Diffusion b-vector (AP direction, first acquisition) |
| │   ├── sub-0001_ses-02_dir-AP_run-01_dwi.json | Diffusion acquisition parameters (AP direction, first acquisition) |
| │   ├── sub-0001_ses-02_dir-AP_run-01_dwi.nii.gz | Diffusion scan (AP direction, first acquisition) |
| │   ├── sub-0001_ses-02_dir-AP_run-02_dwi.bval | Diffusion b-value (AP direction, second acquisition) |
| │   ├── sub-0001_ses-02_dir-AP_run-02_dwi.bvec | Diffusion b-vector (AP direction, second acquisition) |
| │   ├── sub-0001_ses-02_dir-AP_run-02_dwi.json | Diffusion acquisition parameters (AP direction, second acquisition) |
| │   ├── sub-0001_ses-02_dir-AP_run-02_dwi.nii.gz | Diffusion scan (AP direction, second acquisition) |
| │   ├── sub-0001_ses-02_dir-PA_run-01_dwi.bval | Diffusion b-value (PA direction, first acquisition) |
| │   ├── sub-0001_ses-02_dir-PA_run-01_dwi.bvec | Diffusion b-vector (PA direction, first acquisition) |
| │   ├── sub-0001_ses-02_dir-PA_run-01_dwi.json | Diffusion acquisition parameters (PA direction, first acquisition) |
| │   ├── sub-0001_ses-02_dir-PA_run-01_dwi.nii.gz | Diffusion scan (PA direction, first acquisition) |
| │   ├── sub-0001_ses-02_dir-PA_run-02_dwi.bval | Diffusion b-value (PA direction, second acquisition) |
| │   ├── sub-0001_ses-02_dir-PA_run-02_dwi.bvec | Diffusion b-vector (PA direction, second acquisition) |
| │   ├── sub-0001_ses-02_dir-PA_run-02_dwi.json | Diffusion acquisition parameters (PA direction, second acquisition) |
| │   └── sub-0001_ses-02_dir-PA_run-02_dwi.nii.gz | Diffusion scan (PA direction, second acquisition) |
| ├── fmap |  |
| │   ├── sub-0001_ses-02_dir-AP_epi.json | Field map acquisition parameters (AP direction) |
| │   ├── sub-0001_ses-02_dir-AP_epi.nii.gz | Field map scan (AP direction) |
| │   ├── sub-0001_ses-02_dir-PA_epi.json | Field map acquisition parameters (PA direction) |
| │   └── sub-0001_ses-02_dir-PA_epi.nii.gz | Field map scan (PA direction) |
| ├── func |  |
| │   ├── sub-0001_ses-02_task-emotionalconflict_bold.json | t-fMRI acquisition parameters |
| │   ├── sub-0001_ses-02_task-emotionalconflict_bold.nii.gz | t-fMRI scan |
| │   ├── sub-0001_ses-02_task-emotionalconflict_events.json | Data dictionary for sub-0001_ses-02_task-emotionalconflict_events.json |
| │   ├── sub-0001_ses-02_task-emotionalconflict_events.tsv | t-fMRI response data |
| │   ├── sub-0001_ses-02_task-partlycloudy_bold.json | t-fMRI acquisition parameters |
| │   ├── sub-0001_ses-02_task-partlycloudy_bold.nii.gz | t-fMRI scan |
| │   ├── sub-0001_ses-02_task-partlycloudy_events.json | Data dictionary for sub-0001_ses-02_task-partlycloudy_events.tsv |
| │   ├── sub-0001_ses-02_task-partlycloudy_events.tsv | t-fMRI event data |
| │   ├── sub-0001_ses-02_task-rest_dir-AP_bold.json | rs-fMRI acquisition parameters (AP direction) |
| │   ├── sub-0001_ses-02_task-rest_dir-AP_bold.nii.gz | rs-fMRI scan (AP direction) |
| │   ├── sub-0001_ses-02_task-rest_dir-PA_bold.json | rs-fMRI acquisition parameters (PA direction) |
| │   └── sub-0001_ses-02_task-rest_dir-PA_bold.nii.gz | rs-fMRI scan (PA direction) |
| ├── perf |  |
| │   ├── sub-0001_ses-02_asl.json | ASL acquisition parameters |
| │   ├── sub-0001_ses-02_asl.nii.gz | ASL scan |
| │   ├── sub-0001_ses-02_aslcontext.tsv | Volume types of asl scan |
| │   ├── sub-0001_ses-02_m0scan.json | M0 acquisition parameters |
| │   └── sub-0001_ses-02_m0scan.nii.gz | M0 scan |
| └── swi |  |
| ├── sub-0001_ses-02_minIP.json | Minimum intensity projection acquisition parameters |
| ├── sub-0001_ses-02_minIP.nii.gz | Minimum intensity projection scan |
| ├── sub-0001_ses-02_part-mag_GRE.json | Magnitude acquisition parameters |
| ├── sub-0001_ses-02_part-mag_GRE.nii.gz | Magnitude scan |
| ├── sub-0001_ses-02_part-phase_GRE.json | Phase acquisition parameters |
| ├── sub-0001_ses-02_part-phase_GRE.nii.gz | Phase scan |
| ├── sub-0001_ses-02_swi.json | Combined magnitude and phase acquisition parameters |
| └── sub-0001_ses-02_swi.nii.gz | Combined magnitude and phase scan |

## **Supplementary Table 1** Overview of the QTAB OpenNeuro dataset (imaging).

**Bold, purple font** indicates a folder.

*dir* denotes phase encoding direction (*AP* anterior-posterior or *PA* posterior-anterior).

*inv* denotes inversion time (inv-1 or inv-2).

*run* indexes multiple scans acquired in the same session using the same acquisition parameters (e.g. run-01 is the first acquisition, run-02 is the second acquisition).

*ses* denotes session (ses-01 [first session] or ses-02 [second session]).

| Filename | Description/Domain |
| --- | --- |
| non-imaging-phenotypes |  |
| ├── 00_non_imaging_phenotypes_overview.pdf | Description of the items and scales used |
| ├── 01_puberty_ses-01.json | Data dictionary for Puberty data: session 1 |
| ├── 01_puberty_ses-01.tsv | Puberty data: session 1 |
| ├── 01_puberty_ses-02.json | Data dictionary for Puberty data: session 2 |
| ├── 01_puberty_ses-02.tsv | Puberty data: session 2 |
| ├── 02_cognition_ses-01.json | Data dictionary for Cognition data: session 1 |
| ├── 02_cognition_ses-01.tsv | Cognition data: session 1 |
| ├── 02_cognition_ses-02.json | Data dictionary for Cognition data: session 2 |
| ├── 02_cognition_ses-02.tsv | Cognition data: session 2 |
| ├── 03_anxiety_depression_ses-01.json | Data dictionary for Anxiety and/or Depression data: session 1 |
| ├── 03_anxiety_depression_ses-01.tsv | Anxiety and/or Depression data: session 1 |
| ├── 03_anxiety_depression_ses-02.json | Data dictionary for Anxiety and/or Depression data: session 2 |
| ├── 03_anxiety_depression_ses-02.tsv | Anxiety and/or Depression data: session 2 |
| ├── 04_emot_soc_behav_ses-01.json | Data dictionary for Emotional and Social Behaviours data: session 1 |
| ├── 04_emot_soc_behav_ses-01.tsv | Emotional and Social Behaviours data: session 1 |
| ├── 04_emot_soc_behav_ses-02.json | Data dictionary for Emotional and Social Behaviours data: session 2 |
| ├── 04_emot_soc_behav_ses-02.tsv | Emotional and Social Behaviours data: session 2 |
| ├── 05_social_support_family_functioning_ses-01.json | Data dictionary for Social Support and Functioning data: session 1 |
| ├── 05_social_support_family_functioning_ses-01.tsv | Social Support and Functioning data: session 1 |
| ├── 05_social_support_family_functioning_ses-02.json | Data dictionary for Social Support and Functioning data: session 2 |
| ├── 05_social_support_family_functioning_ses-02.tsv | Social Support and Functioning data: session 2 |
| ├── 06_stress_ses-01.json | Data dictionary for Stress data: session 1 |
| ├── 06_stress_ses-01.tsv | Stress data: session 1 |
| ├── 06_stress_ses-02.json | Data dictionary for Stress data: session 2 |
| ├── 06_stress_ses-02.tsv | Stress data: session 2 |
| ├── 07_sleep_physical_health_ses-01.json | Data dictionary for Sleep and Physical Health data: session 1 |
| ├── 07_sleep_physical_health_ses-01.tsv | Sleep and Physical Health data: session 1 |
| ├── 07_sleep_physical_health_ses-02.json | Data dictionary for Sleep and Physical Health data: session 2 |
| ├── 07_sleep_physical_health_ses-02.tsv | Sleep and Physical Health data: session 2 |
| ├── 08_early_life_family_demographics.json | Data dictionary for Early Life and Family Demographics data |
| ├── 08_early_life_family_demographics.tsv | Early Life and Family Demographics data |
| ├── 09_dietary_behaviour_ses-01.json | Data dictionary for Dietary Behaviour data: session 1 |
| ├── 09_dietary_behaviour_ses-01.tsv | Dietary Behaviour data: session 1 |
| ├── 10_covid19.json | Data dictionary for COVID-19 Pandemic Specific Assessments data |
| ├── 10_covid19.tsv | COVID-19 Pandemic Specific Assessments data |
| ├── 11_biological_samples.json | Data dictionary for Biological samples collection statistics |
| ├── 11_biological_samples.tsv | Biological samples collection statistics |
| ├── participants_restricted.json | Data dictionary for participants_restricted.tsv |
| └── participants_restricted.tsv | Participant demographics (age in months, zygosity, zygosity source, multiple birth status, birth order) |

## **Supplementary Table 2** Overview of the QTAB Zenodo dataset (non-imaging phenotypes).

Phenotypic domains (e.g., Puberty, Cognition, Anxiety and/or Depression) are detailed in Table 3 (main text).

**Bold, purple font** indicates a folder.

## **Supplementary Table 3** Overview of questionnaire scale items.

Due to copyright restrictions, scale items from questionnaires are not included in the non-imaging phenotypes dataset. However, we provide detailed instructions for linking item variables to the published questionnaire items (see below). Where necessary, we contacted scale/questionnaire authors to obtain permission to use their measure and share the data collected.

| Puberty | | |
| --- | --- | --- |
| Pubertal Developmental Scale (PDS)  - Carskadon, M. A., & Acebo, C. (1993). A self-administered rating scale for pubertal development. *J Adolesc Health*, *14*(3), 190-195. <https://doi.org/10.1016/1054-139x(93)90004-9>   NOTE: minor version variations of this scale exist.   - We have used the item response options shown in the “Download the Puberty Scale PDF” link found at <http://www.sleepforscience.org/contentmgr/showdetails.php/id/91> - Minor wording changes were undertaken for the parent version (e.g. *For (twin name),* have you noticed…..) | | |
| QTAB Variable | Scale Item Source | Comment |
| PDS01 (self-report)  pPDS01 (parent-report) | Carskadon et al 1993, Table 1, Question 1 | Response Option “has not yet begun to spurt” was changed to “has not yet begun to spurt or grow really fast” as some participants were unfamiliar with the word “spurt”. |
| PDS02  pPDS02 | Carskadon et al 1993, Table 1, Question 2 |  |
| PDS03  pPDS03 | Carskadon et al 1993, Table 1, Question 3 |  |
| PDS04  pPDS04 | Carskadon et al 1993, Table 1  Question 4 FORM FOR BOYS  Question 4 FORM FOR GIRLS |  |
| PDS05  pPDS05 | Carskadon et al 1993, Table 1  Question 5 FORM FOR BOYS  Question 5a FORM FOR GIRLS |  |
| PDS05b  pPDS05b | Carskadon et al 1993, Table 1  Question 5b FORM FOR GIRLS |  |
| Sexual Maturation Scale (SMS)  - Morris, N. M., & Udry, J. R. (1980). Validation of a self-administered instrument to assess stage of adolescent development. *Journal of Youth and Adolescence*, *9*(3), 271-280. <https://doi.org/10.1007/BF02088471> | | |
| QTAB Variable | Scale Item Source | Comment |
| SMS01 (self-report)  pSMS01 (parent-report) | For Girls: Morris et al 1980, Figure 1  For Boys: Morris et al 1980, Figure 4 |  |
| SMS02  pSMS02 | For Girls: Morris et al 1980, Figure 2  For Boys: Morris et al 1980, Figure 3 |  |
| Cognition | | |
| Prospective and Retrospective Memory Questionnaire for Children (PRMQC)  - Talbot, K. D., & Kerns, K. A. (2014). Event- and time-triggered remembering: the impact of attention deficit hyperactivity disorder on prospective memory performance in children. *J Exp Child Psychol*, *127*, 126-143. <https://doi.org/10.1016/j.jecp.2014.02.011>   Note: gender neutral pronous used throughout | | |
| QTAB Variable | Questionnaire Item Source | Comment |
| pPRMQ01 | Talbot et al 2014, Appendix, 1^st^ question |  |
| pPRMQ02 | Talbot et al 2014, Appendix, 2^nd^ question | “he/she” replaced with “they” |
| pPRMQ03 | Talbot et al 2014, Appendix, 3^rd^ question | “he/she” replaced with “they”; “him/her” replaced with “them”; “his/her” replaced with “their” |
| pPRMQ04 | Talbot et al 2014, Appendix, 4^th^ question | “he/she” replaced with “they” |
| pPRMQ05 | Talbot et al 2014, Appendix, 5^th^ question | “he/she” replaced with “they” |
| pPRMQ06 | Talbot et al 2014, Appendix, 6^th^ question |  |
| pPRMQ07 | Talbot et al 2014, Appendix, 7^th^ question | “his/her” replaced with “their” |
| pPRMQ08 | Talbot et al 2014, Appendix, 8^th^ question | “him/her” replaced with “them” |
| pPRMQ09 | Talbot et al 2014, Appendix, 9^th^ question |  |
| pPRMQ10 | Talbot et al 2014, Appendix, 10^th^ question | “him/her” replaced with “them” |
| pPRMQ11 | Talbot et al 2014, Appendix, 11^th^ question | “he/she” replaced with “they”; “his/her” replaced with “their” |
| pPRMQ12 | Talbot et al 2014, Appendix, 12^th^ question | “he/she” replaced with “they” |
| pPRMQ13 | Talbot et al 2014, Appendix, 13^th^ question | “he/she” replaced with “they” |
| pPRMQ14 | Talbot et al 2014, Appendix, 14^th^ question | “he/she” replaced with “they” |
| pPRMQ15 | Talbot et al 2014, Appendix, 15^th^ question | “he/she” replaced with “they” |
| pPRMQ16 | Talbot et al 2014, Appendix, 16^th^ question | “he/she” replaced with “they” |
|  |  |  |
| Empathy Questionnaire for Children and Adolescents (EmQue-CA)  - Overgaauw, S., Rieffe, C., Broekhof, E., Crone, E. A., & Guroglu, B. (2017). Assessing Empathy across Childhood and Adolescence: Validation of the Empathy Questionnaire for Children and Adolescents (EmQue-CA). *Front Psychol*, *8*, 870. <https://doi.org/10.3389/fpsyg.2017.00870> - Variables were presented in the order shown in EmQue-CA_UK.pdf found at https://www.focusonemotions.nl/empathy-questionnaire | | |
| QTAB Variable | Questionnaire Item Source | Comment |
| EmQue1.1 | Overgaauw et al 2017, Table 4, Item 1.1 |  |
| EmQue1.2 | Overgaauw et al 2017, Table 4, Item 1.2 |  |
| EmQue1.3 | Overgaauw et al 2017, Table 4, Item 1.3 |  |
| EmQue1.4 | Overgaauw et al 2017, Table 4, Item 1.4 |  |
| EmQue1.5 | Overgaauw et al 2017, Table 4, Item 1.5 |  |
| EmQue1.6 | Overgaauw et al 2017, Table 4, Item 1.6 |  |
| EmQue2.1 | Overgaauw et al 2017, Table 4, Item 2.1 |  |
| EmQue2.2 | Overgaauw et al 2017, Table 4, Item 2.2 |  |
| EmQue2.3 | Overgaauw et al 2017, Table 4, Item 2.3 | “had” used instead of “has” |
| EmQue3.1 | Overgaauw et al 2017, Table 4, Item 3.1 | “them” used instead of “him” |
| EmQue3.2 | Overgaauw et al 2017, Table 4, Item 3.2 |  |
| EmQue3.3 | Overgaauw et al 2017, Table 4, Item 3.3 |  |
| EmQue3.4 | Overgaauw et al 2017, Table 4, Item 3.4 |  |
| EmQue3.5 | Overgaauw et al 2017, Table 4, Item 3.5 |  |
| Anxiety and/or Depression | | |
| Spence Children’s Anxiety Scale (SCAS)  - Spence, S. H., Barrett, P. M., & Turner, C. M. (2003). Psychometric properties of the Spence Children's Anxiety Scale with young adolescents. *J Anxiety Disord*, *17*(6), 605-625. <https://doi.org/10.1016/s0887-6185(02)00236-0> - The parent-report version is available at https://www.scaswebsite.com/ - there are no filler items in the parent version and so numbering differs from the child self-report scale. The same items are grouped together below.   NOTE: gender neutral pronouns were used in the parent-report version at session 2 (ses-02), but not at session 1 (ses-01) | | |
| QTAB Variable | Scale Item Source | Comment |
| SCAS01 (Self-report)  pSCAS01 (Parent-report) | **Self:** Spence et al 2003, Table 2, Item 1  **Parent:** SCAS website parent version, Item 1 |  |
| SCAS02  pSCAS02 | **Self:** Spence et al 2003, Table 2, Item 2  **Parent:** SCAS website parent version, Item 2 |  |
| SCAS03  pSCAS03 | **Self:** Spence et al 2003, Table 2, Item 3  **Parent:** SCAS website parent version, Item 3 | **Parent-report:**  **ses-01**“s/he” used instead of “my child”  **ses-02 “**s(he)” replaced with “they”; “his/her” replaced with “their” |
| SCAS04  pSCAS04 | **Self:** Spence et al 2003, Table 2, Item 4  **Parent:** SCAS website parent version, Item 4 |  |
| SCAS05  pSCAS05 | **Self:** Spence et al 2003, Table 2, Item 5  **Parent:** SCAS website parent version, Item 5 | **Parent-report:**  **ses-02 “**his/her” replaced with “their” |
| SCAS06  pSCAS06 | **Self:** Spence et al 2003, Table 2, Item 6  **Parent:** SCAS website parent version, Item 6 | **Parent-report:**  **ses-02 “**s(he)” replaced with “they” |
| SCAS07  pSCAS07 | **Self:** Spence et al 2003, Table 2, Item 7  **Parent:** SCAS website parent version, Item 7 | **Parent-report:**  **ses-02 “**s(he)” replaced with “they” |
| SCAS08  pSCAS08 | **Self:** Spence et al 2003, Table 2, Item 8  **Parent:** SCAS website parent version, Item 8 |  |
| SCAS09  pSCAS09 | **Self:** Spence et al 2003, Table 2, Item 9  **Parent:** SCAS website parent version, Item 9 | **Parent-report:**  **ses-02 “**s(he)” replaced with “they”; “him/herself” replaced with “themself” |
| SCAS10  pSCAS10 | **Self:** Spence et al 2003, Table 2, Item 10  **Parent:** SCAS website parent version, Item 10 | **Parent-report:**  **ses-02 “**s(he)” replaced with “they” |
| SCAS11 | Positive filler item – self-report only  SCAS website child version, Item 11 |  |
| SCAS12  pSCAS11 | **Self:** Spence et al 2003, Table 2, Item 12  **Parent:** SCAS website parent version, Item 11 |  |
| SCAS13  pSCAS12 | **Self:** Spence et al 2003, Table 2, Item 13  **Parent:** SCAS website parent version, Item 12 | **Parent-report:**  **ses-02 “**s(he)” replaced with “they” |
| SCAS14  pSCAS13 | **Self:** Spence et al 2003, Table 2, Item 14  **Parent:** SCAS website parent version, Item 13 | **Parent-report:**  **ses-02 “**s(he)” replaced with “they” |
| SCAS15  pSCAS14 | **Self:** Spence et al 2003, Table 2, Item 15  **Parent:** SCAS website parent version, Item 14 | **Parent-report:**  **ses-02 “**s(he)” replaced with “they”; “his/her” replaced with “their” |
| SCAS16  pSCAS15 | **Self:** Spence et al 2003, Table 2, Item 16  **Parent:** SCAS website parent version, Item 15 | **Parent-report:**  **ses-02 “**s(he)” replaced with “they” |
| SCAS17 | Positive filler item – self-report only  SCAS website child version, Item 17 |  |
| SCAS18  pSCAS16 | **Self:** Spence et al 2003, Table 2, Item 18  **Parent:** SCAS website parent version, Item 16 |  |
| SCAS19  pSCAS17 | **Self:** Spence et al 2003, Table 2, Item 19  **Parent:** SCAS website parent version, Item 17 | **Parent-report:**  **ses-02 “**his/her” replaced with “their” |
| SCAS20  pSCAS18 | **Self:** Spence et al 2003, Table 2, Item 20  **Parent:** SCAS website parent version, Item 18 | **Parent-report:**  **ses-02 “**s(he)” replaced with “they”; “his/her” replaced with “their” |
| SCAS21  pSCAS19 | **Self:** Spence et al 2003, Table 2, Item 21  **Parent:** SCAS website parent version, Item 19 |  |
| SCAS22  pSCAS20 | **Self:** Spence et al 2003, Table 2, Item 22  **Parent:** SCAS website parent version, Item 20 | **Parent-report:**  **ses-02 “**him/her” replaced with “them” |
| SCAS23  pSCAS21 | **Self:** Spence et al 2003, Table 2, Item 23  **Parent:** SCAS website parent version, Item 21 |  |
| SCAS24  pSCAS22 | **Self:** Spence et al 2003, Table 2, Item 24  **Parent:** SCAS website parent version, Item 22 | **Parent-report:**  **ses-02 “**s(he)” replaced with “they” |
| SCAS25  pSCAS23 | **Self:** Spence et al 2003, Table 2, Item 25  **Parent:** SCAS website parent version, Item 23 |  |
| SCAS26 | Positive filler item – self-report only  SCAS website child version, Item 26 |  |
| SCAS27  pSCAS24 | **Self:** Spence et al 2003, Table 2, Item 27  **Parent:** SCAS website parent version, Item 24 |  |
| SCAS28  pSCAS25 | **Self:** Spence et al 2003, Table 2, Item 28  **Parent:** SCAS website parent version, Item 25 | **Parent-report:**  **ses-02 “**s(he)” replaced with “they” |
| SCAS29  pSCAS26 | **Self:** Spence et al 2003, Table 2, Item 29  **Parent:** SCAS website parent version, Item 26 | **Parent-report:**  **ses-02 “**him/her” replaced with “them” |
| SCAS30  pSCAS27 | **Self:** Spence et al 2003, Table 2, Item 30  **Parent:** SCAS website parent version, Item 27 |  |
| SCAS31 | Positive filler item -self-report only  SCAS website child version, Item 31 |  |
| SCAS32  pSCAS28 | **Self:** Spence et al 2003, Table 2, Item 32  **Parent:** SCAS website parent version, Item 28 |  |
| SCAS33  pSCAS29 | **Self:** Spence et al 2003, Table 2, Item 33  **Parent:** SCAS website parent version, Item 29 |  |
| SCAS34  pSCAS30 | **Self:** Spence et al 2003, Table 2, Item 34  **Parent:** SCAS website parent version, Item 30 |  |
| SCAS35  pSCAS31 | **Self:** Spence et al 2003, Table 2, Item 35  **Parent:** SCAS website parent version, Item 31 | **Parent-report:**  **ses-02 “**s(he)” replaced with “they” |
| SCAS36  pSCAS32 | **Self:** Spence et al 2003, Table 2, Item 36  **Parent:** SCAS website parent version, Item 32 | **Parent-report:**  **ses-02 “**his/her” replaced with “their” |
| SCAS37  pSCAS33 | **Self:** Spence et al 2003, Table 2, Item 37  **Parent:** SCAS website parent version, Item 33 | **Parent-report:**  **ses-02 “**s(he)” replaced with “they” |
| SCAS38 | Positive filler item – self-report only  SCAS website child version, Item 38 |  |
| SCAS39  pSCAS34 | **Self:** Spence et al 2003, Table 2, Item 39  **Parent:** SCAS website parent version, Item 34 |  |
| SCAS40  pSCAS35 | **Self:** Spence et al 2003, Table 2, Item 40  **Parent:** SCAS website parent version, Item 35 | **Parent-report:**  **ses-02 “**his/her” replaced with “their” |
| SCAS41  pSCAS36 | **Self:** Spence et al 2003, Table 2, Item 41  **Parent:** SCAS website parent version, Item 36 | **Parent-report:**  **ses-02 “**his/her” replaced with “their” |
| SCAS42  pSCAS37 | **Self:** Spence et al 2003, Table 2, Item 42  **Parent:** SCAS website parent version, Item 37 |  |
| SCAS43 | Positive filler item – self-report only  SCAS website child version, Item 43 |  |
| SCAS44  pSCAS38 | **Self:** Spence et al 2003, Table 2, Item 44  **Parent:** SCAS website parent version, Item 38 | **Parent-report:**  **ses-02 “**s(he)” replaced with “they” |
| Short Moods and Feelings Questionnaire (SMFQ)  - Angold, A., Costello, E. J., & Messer, S. C. (1995). Development of a short questionnaire for use in epidemiological studies of depression in children and adolescents. *Int J Methods Psychiatr Res*, *5*, 237-249. - For free download versions of the questionnaire see: <https://devepi.duhs.duke.edu/measures/the-mood-and-feelings-questionnaire-mfq/>   NOTE: gender neutral pronouns were used in the parent-report version at session 2 (ses-02), but not at session 1 (ses-01) | | |
| QTAB Variable | Questionnaire Item Source | Comment |
| SMFQ01 (Self-report)  pSMFQ01 (Parent-report) | Website Child **Self-Report** Short Version, Item 1  Website **Parent-Report** on Child Short Version, Item 1 | **Parent-report:**  **ses-02 “**s/he” replaced with “they” |
| SMFQ02  pSMFQ02 | Website Child **Self-Report** Short Version, Item 2  Website **Parent-Report** on Child Short Version, Item 2 | **Parent-report:**  **ses-02 “**s/he” replaced with “they” |
| SMFQ03  pSMFQ03 | Website Child **Self-Report** Short Version, Item 3  Website **Parent-Report** on Child Short Version, Item 3 | **Parent-report:**  **ses-02 “**s/he” replaced with “they” |
| SMFQ04  pSMFQ04 | Website Child **Self-Report** Short Version, Item 4  Website **Parent-Report** on Child Short Version, Item 4 | **Parent-report:**  **ses-02 “**s/he” replaced with “they” |
| SMFQ05  pSMFQ05 | Website Child **Self-Report** Short Version, Item 5  Website **Parent-Report** on Child Short Version, Item 5 | **Parent-report:**  **ses-02 “**s/he” replaced with “they” |
| SMFQ06  pSMFQ06 | Website Child **Self-Report** Short Version, Item 6  Website **Parent-Report** on Child Short Version, Item 6 | **Parent-report:**  **ses-02 “**s/he” replaced with “they” |
| SMFQ07  pSMFQ07 | Website Child **Self-Report** Short Version, Item 7  Website **Parent-Report** on Child Short Version, Item 7 | **Parent-report:**  **ses-02 “**s/he” replaced with “they” |
| SMFQ08  pSMFQ08 | Website Child **Self-Report** Short Version, Item 8  Website **Parent-Report** on Child Short Version, Item 8 | **Parent-report:**  **ses-02 “**s/he” replaced with “they” |
| SMFQ09  pSMFQ09 | Website Child **Self-Report** Short Version, Item 9  Website **Parent-Report** on Child Short Version, Item 9 | **Parent-report:**  **ses-02 “**s/he” replaced with “they”; “himself/herself” replaced with “themself” |
| SMFQ10  pSMFQ10 | Website Child **Self-Report** Short Version, Item 10  Website **Parent-Report** on Child Short Version, Item 10 | **Parent-report:**  **ses-02 “**s/he” replaced with “they” |
| SMFQ11  pSMFQ11 | Website Child **Self-Report** Short Version, Item 11  Website **Parent-Report** on Child Short Version, Item 11 | **Parent-report:**  **ses-02 “**s/he” replaced with “they”; “him/her” replaced with “them” |
| SMFQ12  pSMFQ12 | Website Child **Self-Report** Short Version, Item 12  Website **Parent-Report** on Child Short Version, Item 12 | **Parent-report:**  **ses-02 “**s/he” replaced with “they” |
| SMFQ13  pSMFQ13 | Website Child **Self-Report** Short Version, Item 13  Website **Parent-Report** on Child Short Version, Item 13 | **Parent-report:**  **ses-02 “**s/he” replaced with “they” |
| Somatic and Psychological Health Report (SPHERE-21)  - Couvy-Duchesne, B., Davenport, T. A., Martin, N. G., Wright, M. J., & Hickie, I. B. (2017). Validation and psychometric properties of the Somatic and Psychological HEalth REport (SPHERE) in a young Australian-based population sample using non-parametric item response theory. *BMC Psychiatry*, *17*(1), 279. <https://doi.org/10.1186/s12888-017-1420-1> - Hickie, I. B., Davenport, T. A., Hadzi-Pavlovic, D., Koschera, A., Naismith, S. L., Scott, E. M., & Wilhelm, K. A. (2001). Development of a simple screening tool for common mental disorders in general practice. *Med J Aust*, *175*(S1), S10-17. <https://doi.org/10.5694/j.1326-5377.2001.tb143784.x> | | |
| QTAB Variable | Report Item Source | Comment |
| SPHERE02 | Couvy-Duchesne et al 2017, Fig 9, Item 2 |  |
| SPHERE03 | Couvy-Duchesne et al 2017, Fig 9, Item 3 |  |
| SPHERE05 | Couvy-Duchesne et al 2017, Fig 9, Item 5 |  |
| SPHERE07 | Couvy-Duchesne et al 2017, Fig 9, Item 7 |  |
| SPHERE08 | Couvy-Duchesne et al 2017, Fig 9, Item 8 |  |
| SPHERE12 | Couvy-Duchesne et al 2017, Fig 9, Item 12 |  |
| SPHERE15 | Couvy-Duchesne et al 2017, Fig 9, Item 15 |  |
| SPHERE17 | Couvy-Duchesne et al 2017, Fig 9, Item 17 |  |
| SPHERE20 | Couvy-Duchesne et al 2017, Fig 9, Item 20 |  |
| SPHERE22 | Couvy-Duchesne et al 2017, Fig 9, Item 22 |  |
| SPHERE23 | Couvy-Duchesne et al 2017, Fig 9, Item 23 |  |
| SPHERE25 | Couvy-Duchesne et al 2017, Fig 9, Item 25 |  |
| SPHERE26 | Couvy-Duchesne et al 2017, Fig 9, Item 26 |  |
| SPHERE27 | Couvy-Duchesne et al 2017, Fig 9, Item 27 |  |
| SPHERE28 | Couvy-Duchesne et al 2017, Fig 9, Item 28 |  |
| SPHERE29 | Couvy-Duchesne et al 2017, Fig 9, Item 29 | We used a longer version of this item “Feeling tired after rest or relaxation?” |
| SPHERE30 | Couvy-Duchesne et al 2017, Fig 9, Item 30 |  |
| SPHERE31 | Couvy-Duchesne et al 2017, Fig 9, Item 31 |  |
| SPHERE32 | Couvy-Duchesne et al 2017, Fig 9, Item 32 |  |
| SPHERE33 | Couvy-Duchesne et al 2017, Fig 9, Item 33 |  |
| SPHERE34 | Couvy-Duchesne et al 2017, Fig 9, Item 34 |  |
| Emotional and Social Behaviours | | |
| Short UPPS-P Impulsive Behaviours Scale in Children (IBS) • Geurten M, et al. Measuring Impulsivity in Children: Adaptation and Validation of a Short Version of the UPPS-P Impulsive Behaviors Scale in Children and Investigation of its Links With ADHD. J Atten Disord. 2021;25(1):105-14. | | |
| QTAB Variable | Scale Item Source | Comment |
| IBS01 | Geurten et al 2021, Appendix A, Item 1 |  |
| IBS02 | Geurten et al 2021, Appendix A, Item 2 |  |
| IBS03 | Geurten et al 2021, Appendix A, Item 3 |  |
| IBS04 | Geurten et al 2021, Appendix A, Item 4 |  |
| IBS05 | Geurten et al 2021, Appendix A, Item 5 |  |
| IBS06 | Geurten et al 2021, Appendix A, Item 6 |  |
| IBS07 | Geurten et al 2021, Appendix A, Item 7 |  |
| IBS08 | Geurten et al 2021, Appendix A, Item 8 |  |
| IBS09 | Geurten et al 2021, Appendix A, Item 9 |  |
| IBS10 | Geurten et al 2021, Appendix A, Item 10 |  |
| IBS11 | Geurten et al 2021, Appendix A, Item 11 |  |
| IBS12 | Geurten et al 2021, Appendix A, Item 12 |  |
| IBS13 | Geurten et al 2021, Appendix A, Item 13 |  |
| IBS14 | Geurten et al 2021, Appendix A, Item 14 |  |
| IBS15 | Geurten et al 2021, Appendix A, Item 15 | “really” was inadvertently left out |
| IBS16 | Geurten et al 2021, Appendix A, Item 16 |  |
| IBS17 | Geurten et al 2021, Appendix A, Item 17 |  |
| IBS18 | Geurten et al 2021, Appendix A, Item 18 |  |
| IBS19 | Geurten et al 2021, Appendix A, Item 19 |  |
| IBS20 | Geurten et al 2021, Appendix A, Item 20 |  |
| Strength and Difficulties Questionnaire (SDQ)  - Goodman, R. (1997). The Strengths and Difficulties Questionnaire: a research note. *J Child Psychol Psychiatry*, *38*(5), 581-586. <https://doi.org/10.1111/j.1469-7610.1997.tb01545.x> - Alternate versions of the SDQ can be found at <https://www.sdqinfo.org/py/sdqinfo/b0.py> – including an “English (Austral)” version for parents or teachers of 11-17 year olds (SDQ_English(Austral)_pt11-17single.pdf) | | |
| QTAB Variable | Questionnaire Item Source | Comment |
| pSDQ01 | Goodman et al 1997, Appendix A, 1^st^ item |  |
| pSDQ02 | Goodman et al 1997, Appendix A, 2^nd^ item |  |
| pSDQ03 | Goodman et al 1997, Appendix A, 3^rd^ item | Minor version difference: “for example, toys, treats, pencils” used instead of “(treats, toys, pencils etc)” |
| pSDQ04 | Goodman et al 1997, Appendix A, 4^th^ item | [www.sdqinfo.org](http://www.sdqinfo.org) Item version used: SDQ_English(Austral)_pt11-17single |
| pSDQ05 | Goodman et al 1997, Appendix A, 5^th^ item |  |
| pSDQ06 | Goodman et al 1997, Appendix A, 6^th^ item |  |
| pSDQ07 | Goodman et al 1997, Appendix A, 7^th^ item |  |
| pSDQ08 | Goodman et al 1997, Appendix A, 8^th^ item |  |
| pSDQ09 | Goodman et al 1997, Appendix A, 9^th^ item |  |
| pSDQ10 | Goodman et al 1997, Appendix A, 10^th^ item |  |
| pSDQ11 | Goodman et al 1997, Appendix A, 11^th^ item |  |
| pSDQ12 | Goodman et al 1997, Appendix A, 12^th^ item | [www.sdqinfo.org](http://www.sdqinfo.org) Item version used: SDQ_English(Austral)_pt11-17single |
| pSDQ13 | Goodman et al 1997, Appendix A, 13^th^ item | [www.sdqinfo.org](http://www.sdqinfo.org) Item version used: SDQ_English(Austral)_pt11-17single |
| pSDQ14 | Goodman et al 1997, Appendix A, 14^th^ item |  |
| pSDQ15 | Goodman et al 1997, Appendix A, 15^th^ item |  |
| pSDQ16 | Goodman et al 1997, Appendix A, 16^th^ item |  |
| pSDQ17 | Goodman et al 1997, Appendix A, 17^th^ item |  |
| pSDQ18 | Goodman et al 1997, Appendix A, 18^th^ item | [www.sdqinfo.org](http://www.sdqinfo.org) Item version used: SDQ_English(Austral)_pt11-17single |
| pSDQ19 | Goodman et al 1997, Appendix A, 19^th^ item |  |
| pSDQ20 | Goodman et al 1997, Appendix A, 20^th^ item |  |
| pSDQ21 | Goodman et al 1997, Appendix A, 21^th^ item |  |
| pSDQ22 | Goodman et al 1997, Appendix A, 22^th^ item |  |
| pSDQ23 | Goodman et al 1997, Appendix A, 23^h^ item |  |
| pSDQ24 | Goodman et al 1997, Appendix A, 24^th^ item |  |
| pSDQ25 | Goodman et al 1997, Appendix A, 25^th^ item | [www.sdqinfo.org](http://www.sdqinfo.org) Item version used: SDQ_English(Austral)_pt11-17single |
| Australian-adapted Hierarchical Personality Inventory for Children (HiPIC-A) Also known as the Australian Child Personality Inventory (ACPI)   - Watt, D., Hopkinson, L., Costello, S., & Roodenburg, J. (2017). Initial Validation and Refinement of the Hierarchical Inventory of Personality for Children in the Australian Context. *Australian Psychologist*, *52*(1), 61-71. <https://doi.org/10.1111/ap.12213> - Hopkinson, L., Watt, D., & Roodenburg, J. (2014). Australian Validation of the Hierarchical Personality Inventory for Children (HiPIC). *The Australian Educational and Developmental Psychologist*, *31*(02), 113-124. <https://doi.org/10.1017/edp.2014.3>   Adapted from:   - Mervielde, I., & De Fruyt, F. (1999). Construction of the Hierarchical Personality Inventory for Children (HiPIC). In I. Mervielde, I. Deary, F. De Fruyt, & F. Ostendorf (Eds.), *Personality psychology in Europe. Proceedings of the Eighth European Conference on Personality Psychology* (pp. 107-111). Tilberg University Press.   NOTE: Scale items are not publicly available (for further information contact [JohnRoodenburg@monash.edu](mailto:JohnRoodenburg@monash.edu)). *Any use of the data must include the above references.*   - Gender neutral pronouns were used for all items. - The QTAB data available are mean domains (factors) and facet scores (range 1-5) as shown below. | | |
| QTAB Variable | Domain/Facet Reference | Comment |
| pHiPICA_amen_score | Domain: Amenability  Watt et al 2017, Table 2, 1^st^ Domain |  |
| pHiPICA_cons_score | Domain: Conscientiousness  Watt et al 2017, Table 2, 2^nd^ Domain |  |
| pHiPICA_emos_score | Domain: Emotional Stability  Watt et al 2017, Table 2, 3^rd^ Domain |  |
| pHiPICA_imag_score | Domain: Imagination  Watt et al 2017, Table 2, 4^th^ Domain |  |
| pHiPICA_extr_score | Domain: Extraversion  Watt et al 2017, Table 2, 5^th^ Domain |  |
| pHiPICA_amenA1_score | Amenability Facet: Irritability  Watt et al 2017, Table 2, A1 |  |
| pHiPICA_amenA2_score | Amenability Facet: Egocentrism  Watt et al 2017, Table 2, A2 |  |
| pHiPICA_amenA3_score | Amenability Facet: Compliance  Watt et al 2017, Table 2, A3 |  |
| pHiPICA_amenA4_score | Amenability Facet: Dominance  Watt et al 2017, Table 2, A4 |  |
| pHiPICA_consC1_score | Conscientiousness Facet: Order  Watt et al 2017, Table 2, C1 |  |
| pHiPICA_consC2_score | Conscientiousness Facet: Achievement/Motivation  Watt et al 2017, Table 2, C2 |  |
| pHiPICA_consC3_score | Conscientiousness Facet: Perseverance  Watt et al 2017, Table 2, C3 |  |
| pHiPICA_consC4_score | Conscientiousness Facet: Concentration  Watt et al 2017, Table 2, C4 |  |
| pHiPICA_emosS1_score | Emotional Stability Facet: Anxiety  Watt et al 2017, Table 2, S1 |  |
| pHiPICA_emosS2_score | Emotional Stability Facet: Self confidence  Watt et al 2017, Table 2, S2 |  |
| pHiPICA_imagI1_score | Imagination Facet: Creativity  Watt et al 2017, Table 2, I1 |  |
| pHiPICA_imagI2_score | Imagination Facet: Curiosity  Watt et al 2017, Table 2, I2 |  |
| pHiPICA_imagI3_score | Imagination Facet: Intellect  Watt et al 2017, Table 2, I3 |  |
| pHiPICA_extrE1_score | Extraversion Facet: Energy  Watt et al 2017, Table 2, E1 |  |
| pHiPICA_extrE2_score | Extraversion Facet: Expressivity  Watt et al 2017, Table 2, E2 |  |
| pHiPICA_extrE3_score | Extraversion Facet: Shyness  Watt et al 2017, Table 2, E3 |  |
| pHiPICA_extrE4_score | Extraversion Facet: Optimism  Watt et al 2017, Table 2, E4 |  |
| Autism Spectrum Quotient – 10 items (AQ-10) (Adolescent)  - Allison, C., Auyeung, B., & Baron-Cohen, S. (2012). Toward brief "Red Flags" for autism screening: The Short Autism Spectrum Quotient and the Short Quantitative Checklist for Autism in toddlers in 1,000 cases and 3,000 controls [corrected]. *J Am Acad Child Adolesc Psychiatry*, *51*(2), 202-212 e207. <https://doi.org/10.1016/j.jaac.2011.11.003>   NOTE: gender neutral pronouns were used. | | |
| QTAB Variable | Scale Item Source | Comment |
| pAQ01 | Allison et al 2012, Table 2, AQ Adolescent, 1^st^ Item | “S/he notices” replaced with “they notice” |
| pAQ02 | Allison et al 2012, Table 2, AQ Adolescent, 2^nd^ Item | “S/he usually concentrates” replaced with “they usually concentrate” |
| pAQ03 | Allison et al 2012, Table 2, AQ Adolescent, 3^rd^ Item | “s/he” replaced with “they” |
| pAQ04 | Allison et al 2012, Table 2, AQ Adolescent, 4^th^ Item | “s/he” replaced with “they” |
| pAQ05 | Allison et al 2012, Table 2, AQ Adolescent, 5^th^ Item | “S/he frequently finds” replaced with “they frequently find”; “s/he doesn’t” replaced with “they don’t” |
| pAQ06 | Allison et al 2012, Table 2, AQ Adolescent, 6^th^ Item | “s/he is” replaced with “they are” |
| pAQ07 | Allison et al 2012, Table 2, AQ Adolescent, 7^th^ Item | “s/he was” replaced with “they were”; “s/he” replaced “they. |
| pAQ08 | Allison et al 2012, Table 2, AQ Adolescent, 8^th^ Item | “s/he finds” replaced with “they find” |
| pAQ09 | Allison et al 2012, Table 2, AQ Adolescent, 9^th^ Item | “s/he finds” replaced with “they find” |
| pAQ10 | Allison et al 2012, Table 2, AQ Adolescent, 10^th^ Item | “s/he finds” replaced with “they find” |
| Children’s Response Styles Questionnaire – rumination subscale (CRSQ)  - Abela, J. R., Brozina, K., & Haigh, E. P. (2002). An examination of the response styles theory of depression in third- and seventh-grade children: a short-term longitudinal study. *J Abnorm Child Psychol*, *30*(5), 515-527. <https://www.ncbi.nlm.nih.gov/pubmed/12403154> - Abela, J. R. Z., Aydin, C. M., & Auerbach, R. P. (2007). Responses to depression in children: reconceptualizing the relation among response styles. *J Abnorm Child Psychol*, *35*, 913-927. <https://doi.org/10.1007/s10802-007-9143-2>   NOTE: The CRSQ consists of 25 items. Data were only collected for the 13 items of the Ruminative Response subscale, which was scored as described in Abela et al 2002. | | |
| QTAB Variable | Questionnaire Item Source | Comment |
| CRSQ01 | Abela et al 2007, Table 1, Item 1 |  |
| CRSQ03 | Abela et al 2007, Table 1, Item 3 |  |
| CRSQ05 | Abela et al 2007, Table 1, Item 5 |  |
| CRSQ07 | Abela et al 2007, Table 1, Item 7 |  |
| CRSQ09 | Abela et al 2007, Table 1, Item 9 |  |
| CRSQ11 | Abela et al 2007, Table 1, Item 11 |  |
| CRSQ13 | Abela et al 2007, Table 1, Item 13 |  |
| CRSQ15 | Abela et al 2007, Table 1, Item 15 |  |
| CRSQ17 | Abela et al 2007, Table 1, Item 17 |  |
| CRSQ19 | Abela et al 2007, Table 1, Item 19 |  |
| CRSQ21 | Abela et al 2007, Table 1, Item 21 |  |
| CRSQ23 | Abela et al 2007, Table 1, Item 23 |  |
| CRSQ25 | Abela et al 2007, Table 1, Item 25 |  |
| Children’s Attributional Style Questionnaire – Revised (CASQ-R)  - Thompson, M., Kaslow, N. J., Weiss, B., & Nolen-Hoeksema, S. (1998). Children's Attributional Style Questionnaire Revised: Psychometric examination. *Psychological Assessment*, *10*(2), 166-170. <https://doi.org/10.1037/1040-3590.10.2.166> | | |
| QTAB Variable | Questionnaire Item Source | Comment |
| CASQ01 | Thompson et al 1998, Appendix, Item 1 |  |
| CASQ02 | Thompson et al 1998, Appendix, Item 2 |  |
| CASQ03 | Thompson et al 1998, Appendix, Item 3 |  |
| CASQ04 | Thompson et al 1998, Appendix, Item 4 |  |
| CASQ05 | Thompson et al 1998, Appendix, Item 5 |  |
| CASQ06 | Thompson et al 1998, Appendix, Item 6 |  |
| CASQ07 | Thompson et al 1998, Appendix, Item 7 |  |
| CASQ08 | Thompson et al 1998, Appendix, Item 8 |  |
| CASQ09 | Thompson et al 1998, Appendix, Item 9 |  |
| CASQ10 | Thompson et al 1998, Appendix, Item 10 |  |
| CASQ11 | Thompson et al 1998, Appendix, Item 11 |  |
| CASQ12 | Thompson et al 1998, Appendix, Item 12 |  |
| CASQ13 | Thompson et al 1998, Appendix, Item 13 |  |
| CASQ14 | Thompson et al 1998, Appendix, Item 14 |  |
| CASQ15 | Thompson et al 1998, Appendix, Item 15 |  |
| CASQ16 | Thompson et al 1998, Appendix, Item 16 |  |
| CASQ17 | Thompson et al 1998, Appendix, Item 17 |  |
| CASQ18 | Thompson et al 1998, Appendix, Item 18 |  |
| CASQ19 | Thompson et al 1998, Appendix, Item 19 |  |
| CASQ20 | Thompson et al 1998, Appendix, Item 20 | “doing” was inadvertently left out  (sentence meaning essentially unchanged) |
| CASQ21 | Thompson et al 1998, Appendix, Item 21 |  |
| CASQ22 | Thompson et al 1998, Appendix, Item 22 |  |
| CASQ23 | Thompson et al 1998, Appendix, Item 23 |  |
| CASQ24 | Thompson et al 1998, Appendix, Item 24 |  |
| Early Adolescent Temperament Questionnaire – Revised (EATQ-R)  - Oldehinkel, A. J., Hartman, C. A., De Winter, A. F., Veenstra, R., & Ormel, J. (2004). Temperament profiles associated with internalizing and externalizing problems in preadolescence. *Dev Psychopathol*, *16*(2), 421-440. <https://www.ncbi.nlm.nih.gov/pubmed/15487604> - Capaldi, D. M., & Rothbart, M. K. (1992). Development and validation of an early adolescent temperament measure. *Journal of Early Adolescence*, *12*(2), 153-173. - Ellis, L. K., & Rothbart, M. K. (2001). Revision of the Early Adolescent Temperament Questionnaire. Poster presented at the 2001 Biennial Meeting of the Society for Research in Child Development, Minneapolis, Minnesota. - The questionnaire is available for research purposes upon request at https://research.bowdoin.edu/rothbart-temperament-questionnaires/ - see Parent-Report   NOTE: Not all subscales were collected at Session 1 (ses-01). Gender neutral pronouns (and corresponding verb) were used at session 2 (ses-02), but not at session 1. | | |
| QTAB Variable | Questionnaire Item Source | Comment |
| pEATQ01 | Bowdoin website parent-report, Item 1 |  |
| pEATQ02 | Bowdoin website parent-report, Item 2 | **ses-02 “**s/he” replaced with “they” |
| pEATQ03 | Bowdoin website parent-report, Item 3 |  |
| pEATQ04 | Bowdoin website parent-report, Item 4 |  |
| pEATQ05 | Bowdoin website parent-report, Item 5 |  |
| pEATQ06 | Bowdoin website parent-report, Item 6 | **ses-02 “**his/her” replaced with “their” |
| pEATQ07 | Bowdoin website parent-report, Item 7 | **ses-02 “**his/her” replaced with “their” |
| pEATQ08 | Bowdoin website parent-report, Item 8 | **ses-02 “**s/he” replaced with “they” |
| pEATQ09 | Bowdoin website parent-report, Item 9 |  |
| pEATQ10 | Bowdoin website parent-report, Item 10 |  |
| pEATQ11 | Bowdoin website parent-report, Item 11 |  |
| pEATQ12 | Bowdoin website parent-report, Item 12 |  |
| pEATQ13 | Bowdoin website parent-report, Item 13 |  |
| pEATQ14 | Bowdoin website parent-report, Item 14 | **ses-02 “**his/her” replaced with “their”; “s/he” replaced with “they” |
| pEATQ15 | Bowdoin website parent-report, Item 15 |  |
| pEATQ16 | Bowdoin website parent-report, Item 16 |  |
| pEATQ17 | Bowdoin website parent-report, Item 17 | **ses-02 “**s/he” replaced with “they” |
| pEATQ18 | Bowdoin website parent-report, Item 18 |  |
| pEATQ19 | Bowdoin website parent-report, Item 19 | **ses-02 “**s/he” replaced with “they” |
| pEATQ20 | Bowdoin website parent-report, Item 20 |  |
| pEATQ21 | Bowdoin website parent-report, Item 21 | **ses-02 “**him/her” replaced with “them” |
| pEATQ22 | Bowdoin website parent-report, Item 22 | **ses-02 “**s/he” replaced with “they” |
| pEATQ23 | Bowdoin website parent-report, Item 23 | **ses-02 “**s/he” replaced with “they”; “her/himself” replaced with “themself” |
| pEATQ24 | Bowdoin website parent-report, Item 24 | **ses-02 “**s/he” replaced with “they” |
| pEATQ25 | Bowdoin website parent-report, Item 25 |  |
| pEATQ26 | Bowdoin website parent-report, Item 26 |  |
| pEATQ27 | Bowdoin website parent-report, Item 27 |  |
| pEATQ28 | Bowdoin website parent-report, Item 28 |  |
| pEATQ29 | Bowdoin website parent-report, Item 29 | **ses-02 “**s/he” replaced with “they” |
| pEATQ30 | Bowdoin website parent-report, Item 30 | **ses-02 “**s/he” replaced with “they” |
| pEATQ31 | Bowdoin website parent-report, Item 31 | **ses-02 “**him/her” replaced with “them”; “s/he” replaced with “they” |
| pEATQ32 | Bowdoin website parent-report, Item 32 |  |
| pEATQ33 | Bowdoin website parent-report, Item 33 |  |
| pEATQ34 | Bowdoin website parent-report, Item 34 |  |
| pEATQ35 | Bowdoin website parent-report, Item 35 |  |
| pEATQ36 | Bowdoin website parent-report, Item 36 | **ses-02 “**his/her” replaced with “their” |
| pEATQ37 | Bowdoin website parent-report, Item 37 |  |
| pEATQ38 | Bowdoin website parent-report, Item 38 |  |
| pEATQ39 | Bowdoin website parent-report, Item 39 | **ses-02 “**him/her” replaced with “them” |
| pEATQ40 | Bowdoin website parent-report, Item 40 |  |
| pEATQ41 | Bowdoin website parent-report, Item 41 |  |
| pEATQ42 | Bowdoin website parent-report, Item 42 |  |
| pEATQ43 | Bowdoin website parent-report, Item 43 |  |
| pEATQ44 | Bowdoin website parent-report, Item 44 |  |
| pEATQ45 | Bowdoin website parent-report, Item 45 | **ses-02 “**s/he” replaced with “they” |
| pEATQ46 | Bowdoin website parent-report, Item 46 |  |
| pEATQ47 | Bowdoin website parent-report, Item 47 | **ses-02 “**him/herself” replaced with “themself” |
| pEATQ48 | Bowdoin website parent-report, Item 48 | **ses-02 “**her/him” replaced with “them” |
| pEATQ49 | Bowdoin website parent-report, Item 49 |  |
| pEATQ50 | Bowdoin website parent-report, Item 50 |  |
| pEATQ51 | Bowdoin website parent-report, Item 51 |  |
| pEATQ52 | Bowdoin website parent-report, Item 52 | **ses-02 “**s/he” replaced with “they”; “her/himself” replaced with “themself” |
| pEATQ53 | Bowdoin website parent-report, Item 53 | **ses-02 “**s/he” replaced with “they” |
| pEATQ54 | Bowdoin website parent-report, Item 54 |  |
| pEATQ55 | Bowdoin website parent-report, Item 55 |  |
| pEATQ56 | Bowdoin website parent-report, Item 56 |  |
| pEATQ57 | Bowdoin website parent-report, Item 57 | **ses-02 “**him/her” replaced with “them” |
| pEATQ58 | Bowdoin website parent-report, Item 58 | **ses-02 “**s/he” replaced with “they”; “her/his” replaced with “their” |
| pEATQ59 | Bowdoin website parent-report, Item 59 | **ses-02 “**his/her” replaced with “their” |
| pEATQ60 | Bowdoin website parent-report, Item 60 |  |
| pEATQ61 | Bowdoin website parent-report, Item 61 |  |
| pEATQ62 | Bowdoin website parent-report, Item 62 |  |
| Social Support and Family Functioning | | |
| Multidimensional Scale of Perceived Social Support (MSPSS)  - Zimet, G. D., Dahlem, N. W., Zimet, S. G., & Farley, G. K. (1988). The Multidimensional Scale of Perceived Social Support. *Journal of Personality Assessment*, *52*(1), 30-41. <https://doi.org/10.1207/s15327752jpa5201_2> | | |
| QTAB Variable | Scale Item Source | Comment |
| MSPSS01 | Zimet et al 1988, Table 1, Item 1 |  |
| MSPSS02 | Zimet et al 1988, Table 1, Item 2 |  |
| MSPSS03 | Zimet et al 1988, Table 1, Item 3 |  |
| MSPSS04 | Zimet et al 1988, Table 1, Item 4 |  |
| MSPSS05 | Zimet et al 1988, Table 1, Item 5 |  |
| MSPSS06 | Zimet et al 1988, Table 1, Item 6 |  |
| MSPSS07 | Zimet et al 1988, Table 1, Item 7 |  |
| MSPSS08 | Zimet et al 1988, Table 1, Item 8 |  |
| MSPSS09 | Zimet et al 1988, Table 1, Item 9 |  |
| MSPSS10 | Zimet et al 1988, Table 1, Item 10 |  |
| MSPSS11 | Zimet et al 1988, Table 1, Item 11 |  |
| MSPSS12 | Zimet et al 1988, Table 1, Item 12 |  |
| Alabama Parenting Questionnaire (APQ)  - Shelton, K. K., Frick, P. J., & Wootton, J. (1996). Assessment of parenting practices in families of elementary school-age children. *Journal of Clinical Child Psychology*, *25*(3), 317-329. <https://doi.org/DOI> 10.1207/s15374424jccp2503_8   NOTE: Not all subscales were collected at session 1 (only Poor Monitoring/Supervision) or session 2 (only Involvement, Positive Parenting, and Poor Monitoring/Supervision). Minor wording changes were made as questions are posed for more than one child (e.g. “child” is replaced with “children”; “his/her” is replaced with “their” etc.). Parent responds separately for each child. | | |
| QTAB Variable | Questionnaire Item Source | Comment |
| pAPQ01 | Shelton et al 1996, Table 2, Involvement, Item 1 |  |
| pAPQ02 | Shelton et al 1996, Table 2, Positive Parenting, Item 2 | “is doing a good job with something” inadvertently replaced with “do something well” |
| pAPQ04 | Shelton et al 1996, Table 2, Involvement, Item 4 |  |
| pAPQ05 | Shelton et al 1996, Table 2, Positive Parenting, Item 5 |  |
| pAPQ06 | Shelton et al 1996, Table 2, Poor Monitoring, Item 6 |  |
| pAPQ07 | Shelton et al 1996, Table 2, Involvement, Item 7 |  |
| pAPQ09 | Shelton et al 1996, Table 2, Involvement, Item 9 |  |
| pAPQ10 | Shelton et al 1996, Table 2, Poor Monitoring, Item 10 |  |
| pAPQ11 | Shelton et al 1996, Table 2, Involvement, Item 11 |  |
| pAPQ13 | Shelton et al 1996, Table 2, Positive Parenting, Item 13 |  |
| pAPQ14 | Shelton et al 1996, Table 2, Involvement, Item 14 |  |
| pAPQ15 | Shelton et al 1996, Table 2, Involvement, Item 15 | “drive” replaced with “take” as some families use other means of transport |
| pAPQ16 | Shelton et al 1996, Table 2, Positive Parenting, Item 16 |  |
| pAPQ17 | Shelton et al 1996, Table 2, Poor Monitoring, Item 17 |  |
| pAPQ18 | Shelton et al 1996, Table 2, Positive Parenting, Item 18 |  |
| pAPQ19 | Shelton et al 1996, Table 2, Poor Monitoring, Item 19 |  |
| pAPQ20 | Shelton et al 1996, Table 2, Involvement, Item 20 |  |
| pAPQ21 | Shelton et al 1996, Table 2, Poor Monitoring, Item 21 |  |
| pAPQ23 | Shelton et al 1996, Table 2, Involvement, Item 23 |  |
| pAPQ24 | Shelton et al 1996, Table 2, Poor Monitoring, Item 24 |  |
| pAPQ26 | Shelton et al 1996, Table 2, Involvement, Item 26 |  |
| pAPQ27 | Shelton et al 1996, Table 2, Positive Parenting, Item 27 |  |
| pAPQ28 | Shelton et al 1996, Table 2, Poor Monitoring, Item 28 |  |
| pAPQ29 | Shelton et al 1996, Table 2, Poor Monitoring, Item 29 |  |
| pAPQ30 | Shelton et al 1996, Table 2, Poor Monitoring, Item 30 |  |
| pAPQ32 | Shelton et al 1996, Table 2, Poor Monitoring, Item 32 |  |
| McMaster Family Assessment Device (FAD)  - Epstein, N. B., Baldwin, L. M., & Bishop, D. S. (1983). THE McMASTER FAMILY ASSESSMENT DEVICE*. *Journal of Marital and Family Therapy*, *9*(2), 171-180. <https://doi.org/10.1111/j.1752-0606.1983.tb01497.x> - Kabacoff, R. I., Miller, I. W., Bishop, D. S., Epstein, N. B., & Keitner, G. I. (1990). A psychometric study of the McMaster Family Assessment Device in psychiatric, medical, and nonclinical samples. *Journal of Family Psychology*, *3*(4), 431-439. <https://doi.org/10.1037/h0080547>   *For more about the 12-item General Functioning subscale see*   - Boterhoven de Haan, K. L., Hafekost, J., Lawrence, D., Sawyer, M. G., & Zubrick, S. R. (2015). Reliability and validity of a short version of the general functioning subscale of the McMaster Family Assessment Device. *Fam Process*, *54*(1), 116-123. <https://doi.org/10.1111/famp.12113>   NOTE: the 6-time subscale used in Boterhoven de Haan et al 2015 is a match to QTAB items pFAD06, pFAD16, pFAD26, pFAD36, pFAD46, pFAD56.  *These items were reverse scored (note that we were unable to obtain official identification of items to be reverse scored). | | |
| QTAB Variable | Device Item Source | Comment |
| pFAD01* | Buckland Thesis, Appendix 3, Item 1  Epstein et al 1983, Table 1, General, 1^st^ Item |  |
| pFAD02 | Buckland Thesis, Appendix 3, Item 2 | This item contributed to the “Problem Solving” Scale (personal communication with Sharon Buckland) |
| pFAD03 | Buckland Thesis, Appendix 3, Item 3  Epstein et al 1983, Table 1, Communication, 1^st^ Item |  |
| pFAD04* | Buckland Thesis, Appendix 3, Item 4  Epstein et al 1983, Table 1, Roles, 1^st^ Item |  |
| pFAD05* | Buckland Thesis, Appendix 3, Item 5  Epstein et al 1983, Table 1, Affect Involvement, 1^st^ Item |  |
| pFAD06 | Buckland Thesis, Appendix 3, Item 6  Epstein et al 1983, Table 1, General, 2^nd^ Item |  |
| pFAD07* | Buckland Thesis, Appendix 3, Item 7  Epstein et al 1983, Table 1, Behavior Control, 1^st^ Item |  |
| pFAD08* | Buckland Thesis, Appendix 3, Item 8 | This item contributed to the “Roles” Scale (personal communication with Sharon Buckland) |
| pFAD09* | Buckland Thesis, Appendix 3, Item 9  Epstein et al 1983, Table 1, Affect Resp, 1^st^ Item |  |
| pFAD10 | Buckland Thesis, Appendix 3, Item 10  Epstein et al 1983, Table 1, Roles, 2^nd^ Item |  |
| pFAD11* | Buckland Thesis, Appendix 3, Item 11  Epstein et al 1983, Table 1, General, 3^rd^ Item |  |
| pFAD12 | Buckland Thesis, Appendix 3, Item 12  Epstein et al 1983, Table 1, Problem Solving, 1^st^ Item |  |
| pFAD13* | Buckland Thesis, Appendix 3, Item 13  Epstein et al 1983, Table 1, Affect Involvement, 2^nd^ Item |  |
| pFAD14* | Buckland Thesis, Appendix 3, Item 14  Epstein et al 1983, Table 1, Communication, 2^nd^ Item |  |
| pFAD15* | Buckland Thesis, Appendix 3, Item 15  Epstein et al 1983, Table 1, Roles, 3^rd^ Item |  |
| pFAD16 | Buckland Thesis, Appendix 3, Item 16  Epstein et al 1983, Table 1, General, 4^th^ Item |  |
| pFAD17* | Buckland Thesis, Appendix 3, Item 17  Epstein et al 1983, Table 1, Behavior Control, 2^nd^ Item |  |
| pFAD18 | Buckland Thesis, Appendix 3, Item 18  Epstein et al 1983, Table 1, Communication, 3^rd^ Item |  |
| pFAD19* | Buckland Thesis, Appendix 3, Item 19  Epstein et al 1983, Table 1, Affect Resp, 2^nd^ Item |  |
| pFAD20 | Buckland Thesis, Appendix 3, Item 20  Epstein et al 1983, Table 1, Behavior Control, 3^rd^ Item |  |
| pFAD21* | Buckland Thesis, Appendix 3, Item 21  Epstein et al 1983, Table 1, General, 5^th^ Item |  |
| pFAD22* | Buckland Thesis, Appendix 3, Item 22 | This item contributed to the “Communication” Scale (personal communication with Sharon Buckland) |
| pFAD23* | Buckland Thesis, Appendix 3, Item 23  Epstein et al 1983, Table 1, Roles, 4^th^ Item | Wording as per Epstein et al 1983 |
| pFAD24 | Buckland Thesis, Appendix 3, Item 24  Epstein et al 1983, Table 1, Problem Solving, 2^nd^ Item |  |
| pFAD25* | Buckland Thesis, Appendix 3, Item 25  Epstein et al 1983, Table 1, Affect Involvement, 3^rd^ Item |  |
| pFAD26 | Buckland Thesis, Appendix 3, Item 26  Epstein et al 1983, Table 1, General, 6^th^ Item |  |
| pFAD27* | Buckland Thesis, Appendix 3, Item 27  Epstein et al 1983, Table 1, Behavior Control, 4^th^ Item |  |
| pFAD28* | Buckland Thesis, Appendix 3, Item 28  Epstein et al 1983, Table 1, Affect Resp, 3^rd^ Item |  |
| pFAD29 | Buckland Thesis, Appendix 3, Item 29 | This item contributed to the “Communication” Scale (personal communication with Sharon Buckland) |
| pFAD30 | Buckland Thesis, Appendix 3, Item 30 | This item contributed to the “Roles” Scale (personal communication with Sharon Buckland) |
| pFAD31* | Buckland Thesis, Appendix 3, Item 31  Epstein et al 1983, Table 1, General, 7^th^ Item |  |
| pFAD32 | Buckland Thesis, Appendix 3, Item 32  Epstein et al 1983, Table 1, Behavior control, 5^th^ Item |  |
| pFAD33* | Buckland Thesis, Appendix 3, Item 33  Epstein et al 1983, Table 1, Affect Involvement, 4^th^ Item |  |
| pFAD34* | Buckland Thesis, Appendix 3, Item 34  Epstein et al 1983, Table 1, Roles, 5^th^ Item |  |
| pFAD35* | Buckland Thesis, Appendix 3, Item 35 | This item contributed to the “Communication” Scale (personal communication with Sharon Buckland) |
| pFAD36 | Buckland Thesis, Appendix 3, Item 36  Epstein et al 1983, Table 1, General, 8^th^ Item |  |
| pFAD37* | Buckland Thesis, Appendix 3, Item 37  Epstein et al 1983, Table 1, Affect Involvement, 5^th^ Item |  |
| pFAD38 | Buckland Thesis, Appendix 3, Item 38  Epstein et al 1983, Table 1, Problem Solving, 3^rd^ Item |  |
| pFAD39* | Buckland Thesis, Appendix 3, Item 39  Epstein et al 1983, Table 1, Affect Resp, 4^th^ Item |  |
| pFAD40 | Buckland Thesis, Appendix 3, Item 40  Epstein et al 1983, Table 1, Roles, 6^th^ Item |  |
| pFAD41* | Buckland Thesis, Appendix 3, Item 41  Epstein et al 1983, Table 1, General, 9^th^ Item |  |
| pFAD42* | Buckland Thesis, Appendix 3, Item 42  Epstein et al 1983, Table 1, Affect Involvement, 6^th^ Item |  |
| pFAD43 | Buckland Thesis, Appendix 3, Item 43  Epstein et al 1983, Table 1, Communication, 4^th^ Item | Wording as per Epstein et al 1983 |
| pFAD44* | Buckland Thesis, Appendix 3, Item 44  Epstein et al 1983, Table 1, Behavior Control, 6^th^ Item |  |
| pFAD45* | Buckland Thesis, Appendix 3, Item 45  Epstein et al 1983, Table 1, Roles, 7^th^ Item |  |
| pFAD46 | Buckland Thesis, Appendix 3, Item 46  Epstein et al 1983, Table 1, General, 10^th^ Item |  |
| pFAD47* | Buckland Thesis, Appendix 3, Item 47  Epstein et al 1983, Table 1, Behavior control, 7^th^ Item |  |
| pFAD48* | Buckland Thesis, Appendix 3, Item 48  Epstein et al 1983, Table 1, Behavior Control, 7^th^ Item |  |
| pFAD49 | Buckland Thesis, Appendix 3, Item 49  Epstein et al 1983, Table 1, Affect Resp, 5^th^ Item |  |
| pFAD50 | Buckland Thesis, Appendix 3, Item 50  Epstein et al 1983, Table 1, Problem Solving, 4^th^ Item |  |
| pFAD51* | Buckland Thesis, Appendix 3, Item 51  Epstein et al 1983, Table 1, General, 11^th^ Item |  |
| pFAD52* | Buckland Thesis, Appendix 3, Item 52  Epstein et al 1983, Table 1, Communication, 5^th^ Item |  |
| pFAD53* | Buckland Thesis, Appendix 3, Item 53  Epstein et al 1983, Table 1, Roles, 8^th^ Item |  |
| pFAD54* | Buckland Thesis, Appendix 3, Item 54  Epstein et al 1983, Table 1, Affect Involvement, 7^th^ Item |  |
| pFAD55 | Buckland Thesis, Appendix 3, Item 55  Epstein et al 1983, Table 1, Behavior Control, 9^th^ Item |  |
| pFAD56 | Buckland Thesis, Appendix 3, Item 56  Epstein et al 1983, Table 1, General, 12^th^ Item |  |
| pFAD57 | Buckland Thesis, Appendix 3, Item 57  Epstein et al 1983, Table 1, Affect Resp, 6^th^ Item |  |
| pFAD58* | Buckland Thesis, Appendix 3, Item 58 | This item contributed to the “Roles” Scale (personal communication with Sharon Buckland) |
| pFAD59 | Buckland Thesis, Appendix 3, Item 59  Epstein et al 1983, Table 1, Communication, 6^th^ Item |  |
| pFAD60 | Buckland Thesis, Appendix 3, Item 60  Epstein et al 1983, Table 1, Problem Solving, 5^th^ Item |  |
| Stress | | |
| Daily Life Stressors Scale (DLSS)  - Kearney, C. A., Drabman, R. S., & Beasley, J. F. (1993). The trials of childhood: the development, reliability, and validity of the Daily Life Stressors Scale. *J Child Fam Stud*, *2*(4), 371-388. <https://doi.org/10.1007/BF01321232> | | |
| QTAB Variable | Scale Item Source | Comment |
| DLSS01 | Kearney et al 1993, Table 1, Item 1 |  |
| DLSS02 | Kearney et al 1993, Table 1, Item 2 |  |
| DLSS03 | Kearney et al 1993, Table 1, Item 3 |  |
| DLSS04 | Kearney et al 1993, Table 1, Item 4 |  |
| DLSS05 | Kearney et al 1993, Table 1, Item 5 |  |
| DLSS06 | Kearney et al 1993, Table 1, Item 6 |  |
| DLSS07 | Kearney et al 1993, Table 1, Item 7 |  |
| DLSS08 | Kearney et al 1993, Table 1, Item 8 |  |
| DLSS09 | Kearney et al 1993, Table 1, Item 9 |  |
| DLSS10 | Kearney et al 1993, Table 1, Item 10 |  |
| DLSS11 | Kearney et al 1993, Table 1, Item 11 |  |
| DLSS12 | Kearney et al 1993, Table 1, Item 12 |  |
| DLSS13 | Kearney et al 1993, Table 1, Item 13 |  |
| DLSS14 | Kearney et al 1993, Table 1, Item 14 |  |
| DLSS15 | Kearney et al 1993, Table 1, Item 15 |  |
| DLSS16 | Kearney et al 1993, Table 1, Item 16 |  |
| DLSS17 | Kearney et al 1993, Table 1, Item 17 |  |
| DLSS18 | Kearney et al 1993, Table 1, Item 18 |  |
| DLSS19 | Kearney et al 1993, Table 1, Item 19 |  |
| DLSS20 | Kearney et al 1993, Table 1, Item 20 |  |
| DLSS21 | Kearney et al 1993, Table 1, Item 21 |  |
| DLSS22 | Kearney et al 1993, Table 1, Item 22 |  |
| DLSS23 | Kearney et al 1993, Table 1, Item 23 |  |
| DLSS24 | Kearney et al 1993, Table 1, Item 24 |  |
| DLSS25 | Kearney et al 1993, Table 1, Item 25 |  |
| DLSS26 | Kearney et al 1993, Table 1, Item 26 |  |
| DLSS27 | Kearney et al 1993, Table 1, Item 27 |  |
| DLSS28 | Kearney et al 1993, Table 1, Item 28 |  |
| DLSS29 | Kearney et al 1993, Table 1, Item 29 |  |
| DLSS30 | Kearney et al 1993, Table 1, Item 30 |  |
| Gatehouse Bullying Scale (GBS)  - Bond, L., Wolfe, S., Tollit, M., Butler, H., & Patton, G. (2007). A comparison of the Gatehouse Bullying Scale and the peer relations questionnaire for students in secondary school. *J Sch Health*, *77*(2), 75-79. <https://doi.org/10.1111/j.1746-1561.2007.00170.x>   Data were scored as described in:   - Thomas, H. J., Chan, G. C., Scott, J. G., Connor, J. P., Kelly, A. B., & Williams, J. (2016). Association of different forms of bullying victimisation with adolescents' psychological distress and reduced emotional wellbeing. *Aust N Z J Psychiatry*, *50*(4), 371-379. <https://doi.org/10.1177/0004867415600076> | | |
| QTAB Variable | Scale Item Source | Comment |
| GBS01a | Bond et al 2007, Table 1, GBS, Item 1a |  |
| GBS01b | Bond et al 2007, Table 1, GBS, Item 1b |  |
| BGS01c | Bond et al 2007, Table 1, GBS, Item 1c |  |
| GBS02a | Bond et al 2007, Table 1, GBS, Item 2a |  |
| GBS02b | Bond et al 2007, Table 1, GBS, Item 2b |  |
| BGS02c | Bond et al 2007, Table 1, GBS, Item 2c |  |
| GBS03a | Bond et al 2007, Table 1, GBS, Item 3a |  |
| GBS03b | Bond et al 2007, Table 1, GBS, Item 3b |  |
| BGS03c | Bond et al 2007, Table 1, GBS, Item 3c |  |
| GBS04a | Bond et al 2007, Table 1, GBS, Item 4a |  |
| GBS04b | Bond et al 2007, Table 1, GBS, Item 4b |  |
| BGS04c | Bond et al 2007, Table 1, GBS, Item 4c |  |
| Childhood Life Events Questionnaire (CLEQ)  - Upthegrove, R., Chard, C., Jones, L., Gordon-Smith, K., Forty, L., Jones, I., & Craddock, N. (2015). Adverse childhood events and psychosis in bipolar affective disorder. *Br J Psychiatry*, *206*(3), 191-197. <https://doi.org/10.1192/bjp.bp.114.152611>   Note: The CLEQ was used in questionnaire format and was answered by a parent for each adolescent twin. Each item was prefaced with “Have your children experienced: …”. | | |
| QTAB Variable | Questionnaire Item Source | Comment |
| pCLEQ01 | Upthegrove et al 2015, Data Supplement, Item 1 |  |
| pCLEQ01a | If yes to Item 1, at what age? |  |
| pCLEQ02 | Upthegrove et al 2015, Data Supplement, Item 2 |  |
| pCLEQ02a | If yes to Item 2, at what age? |  |
| pCLEQ03 | Upthegrove et al 2015, Data Supplement, Item 3 |  |
| pCLEQ03a | If yes to Item 3, at what age? |  |
| pCLEQ04 | Upthegrove et al 2015, Data Supplement, Item 4 |  |
| pCLEQ04a | If yes to Item 4, at what age? |  |
| pCLEQ05 | Upthegrove et al 2015, Data Supplement, Item 5 |  |
| pCLEQ05a | If yes to Item 5, at what age? |  |
| pCLEQ06 | Upthegrove et al 2015, Data Supplement, Item 6 |  |
| pCLEQ06a | If yes to Item 6, at what age? |  |
| pCLEQ07 | Upthegrove et al 2015, Data Supplement, Item 7 |  |
| pCLEQ07a | If yes to Item 7, at what age? |  |
| pCLEQ08 | Upthegrove et al 2015, Data Supplement, Item 8 |  |
| pCLEQ08a | If yes to Item 8, at what age? |  |
| pCLEQ09 | Upthegrove et al 2015, Data Supplement, Item 9 |  |
| pCLEQ09a | If yes to Item 9, at what age? |  |
| pCLEQ10 | Upthegrove et al 2015, Data Supplement, Item 10 |  |
| pCLEQ10a | If yes to Item 10, at what age? |  |
|  | Upthegrove et al 2015, Data Supplement, Item 11 | This item was not included. |
| pCLEQ12 | Upthegrove et al 2015, Data Supplement, Item 12 |  |
| pCLEQ12a | If yes to Item 12, at what age? |  |
|  | Upthegrove et al 2015, Data Supplement, Item 13 | This item not available. |
| Prenatal Stress Exposure Scale (PSES) Adapted from interview reported in   - Favaro, A., Tenconi, E., Degortes, D., Manara, R., & Santonastaso, P. (2015). Neural correlates of prenatal stress in young women. *Psychol Med*, *45*(12), 2533-2543. <https://doi.org/10.1017/S003329171500046X> | | |
| QTAB Variable | Scale Items  (Were any of the following traumatic or stressful events experienced **in the 12 months before the twin’s conception, during the pregnancy, or in the first months after the birth of the twins**?) | Comment |
| pPSES01a | Did this occur: Accidents (including those of a close friend or relative) |  |
| pPSES01b | If yes, when did the event occur (can select multiple)? | - In 12 months before conception - During the pregnancy - After delivery |
| pPSES01c | If yes, what level of stress did the (most stressful) event cause? | - No significant stress - Some stress - Moderate stress - Substantial stress - Extreme stress |
| pPSES02a | Did this occur: Death of a friend or close relative |  |
| pPSES02b | If yes, when did the event occur (can select multiple)? |  |
| pPSES02c | If yes, what level of stress did the (most stressful) event cause? |  |
| pPSES03a | Did this occur: Health problems (unrelated to the pregnancy) |  |
| pPSES03b | If yes, when did the event occur (can select multiple)? |  |
| pPSES03c | If yes, what level of stress did the (most stressful) event cause? |  |
| pPSES04a | Did this occur: Severe health problem of a close friend or relative, or need to give assistance to a sick friend or relative |  |
| pPSES04b | If yes, when did the event occur (can select multiple)? |  |
| pPSES04c | If yes, what level of stress did the (most stressful) event cause? |  |
| pPSES05a | Did this occur: Natural disasters (e.g. flooding) |  |
| pPSES05b | If yes, when did the event occur (can select multiple)? |  |
| pPSES05c | If yes, what level of stress did the (most stressful) event cause? |  |
| pPSES06a | Did this occur: Severe interpersonal conflicts with a partner or close relative |  |
| pPSES06b | If yes, when did the event occur (can select multiple)? |  |
| pPSES06c | If yes, what level of stress did the (most stressful) event cause? |  |
| pPSES07a | Did this occur: Separation from a partner |  |
| pPSES07b | If yes, when did the event occur (can select multiple)? |  |
| pPSES07c | If yes, what level of stress did the (most stressful) event cause? |  |
| pPSES08a | Did this occur: Severe legal or economic problems |  |
| pPSES08b | If yes, when did the event occur (can select multiple)? |  |
| pPSES08c | If yes, what level of stress did the (most stressful) event cause? |  |
| pPSES09a | Did this occur: Relocation (i.e. moving house) |  |
| pPSES09b | If yes, when did the event occur (can select multiple)? |  |
| pPSES09c | If yes, what level of stress did the (most stressful) event cause? |  |
| pPSES10a | Did this occur: Personal violence |  |
| pPSES10b | If yes, when did the event occur (can select multiple)? |  |
| pPSES10c | If yes, what level of stress did the (most stressful) event cause? |  |
| pPSES11a | Did this occur: Sexual abuse or maltreatment |  |
| pPSES11b | If yes, when did the event occur (can select multiple)? |  |
| pPSES11c | If yes, what level of stress did the (most stressful) event cause? |  |
| pPSES12a | Did this occur: Miscarriage or abortion (occurring in the 12 months before the twin’s conception) |  |
| pPSES12c | If yes, what level of stress did the (most stressful) event cause? |  |
| pPSES13a | Did this occur: Other offspring death |  |
| pPSES13b | If yes, when did the event occur (can select multiple)? |  |
| pPSES13c | If yes, what level of stress did the (most stressful) event cause? |  |
| pPSES14a | Did this occur: Other traumatic or stressful event |  |
| pPSES14b | If yes, when did the event occur (can select multiple)? |  |
| pPSES14c | If yes, what level of stress did the (most stressful) event cause? |  |
| Parental Stress Scale (PaSS)  - Berry, J. O., & Jones, W. H. (1995). The Parental Stress Scale: initial psychometric evidence. *Journal of Social and Personal Relationships*, *12*(3), 463-472. <https://doi.org/10.1177/0265407595123009> | | |
| QTAB Variable | Scale Item Source | Comment |
| pPaSS01 | Berry et al 1995, Table 1, Item 1 |  |
| pPaSS02 | Berry et al 1995, Table 1, Item 2 |  |
| pPaSS03 | Berry et al 1995, Table 1, Item 3 |  |
| pPaSS04 | Berry et al 1995, Table 1, Item 4 |  |
| pPaSS05 | Berry et al 1995, Table 1, Item 5 |  |
| pPaSS06 | Berry et al 1995, Table 1, Item 6 |  |
| pPaSS07 | Berry et al 1995, Table 1, Item 7 |  |
| pPaSS08 | Berry et al 1995, Table 1, Item 8 |  |
| pPaSS09 | Berry et al 1995, Table 1, Item 9 |  |
| pPaSS10 | Berry et al 1995, Table 1, Item 10 |  |
| pPaSS11 | Berry et al 1995, Table 1, Item 11 |  |
| pPaSS12 | Berry et al 1995, Table 1, Item 12 |  |
| pPaSS13 | Berry et al 1995, Table 1, Item 13 |  |
| pPaSS14 | Berry et al 1995, Table 1, Item 14 |  |
| pPaSS15 | Berry et al 1995, Table 1, Item 15 |  |
| pPaSS16 | Berry et al 1995, Table 1, Item 16 |  |
| pPaSS17 | Berry et al 1995, Table 1, Item 17 |  |
| pPaSS18 | Berry et al 1995, Table 1, Item 18 |  |
| List of Threatening Experiences (LTE)  - Motrico, E., Moreno-Kustner, B., de Dios Luna, J., Torres-Gonzalez, F., King, M., Nazareth, I., Monton-Franco, C., Gilde Gomez-Barragan, M. J., Sanchez-Celaya, M., Diaz-Barreiros, M. A., Vicens, C., Moreno-Peral, P., & Bellon, J. A. (2013). Psychometric properties of the List of Threatening Experiences--LTE and its association with psychosocial factors and mental disorders according to different scoring methods. *J Affect Disord*, *150*(3), 931-940. <https://doi.org/10.1016/j.jad.2013.05.017>   NOTE: all items are prefaced with “during the last 12 months, have you experienced…” | | |
| QTAB Variable | Scale Item Source | Comment |
| pLTE01 | Motrico et al 2013, Table 2, Item 1 |  |
| pLTE02 | Motrico et al 2013, Table 2, Item 2 |  |
| pLTE03 | Motrico et al 2013, Table 2, Item 3 |  |
| pLTE04 | Motrico et al 2013, Table 2, Item 4 |  |
| pLTE05 | Motrico et al 2013, Table 2, Item 5 |  |
| pLTE06 | Motrico et al 2013, Table 2, Item 6 |  |
| pLTE07 | Motrico et al 2013, Table 2, Item 7 |  |
| pLTE08 | Motrico et al 2013, Table 2, Item 8 |  |
| pLTE09 | Motrico et al 2013, Table 2, Item 9 |  |
| pLTE10 | Motrico et al 2013, Table 2, Item 10 |  |
| pLTE11 | Motrico et al 2013, Table 2, Item 11 |  |
| pLTE12 | Motrico et al 2013, Table 2, Item 12 |  |
| Sleep and Physical Health | | |
| Pediatric Daytime Sleepiness Scale (PDSS)  - Drake, C., Nickel, C., Burduvali, E., Roth, T., Jefferson, C., & Pietro, B. (2003). The pediatric daytime sleepiness scale (PDSS): sleep habits and school outcomes in middle-school children. *Sleep*, *26*(4), 455-458. <https://www.ncbi.nlm.nih.gov/pubmed/12841372> | | |
| QTAB Variable | Scale Item Source | Comment |
| PDSS01 | Drake et al 2003, Appendix, Item 1 |  |
| PDSS02 | Drake et al 2003, Appendix, Item 2 |  |
| PDSS03 | Drake et al 2003, Appendix, Item 3 |  |
| PDSS04 | Drake et al 2003, Appendix, Item 4 |  |
| PDSS05 | Drake et al 2003, Appendix, Item 5 |  |
| PDSS06 | Drake et al 2003, Appendix, Item 6 |  |
| PDSS07 | Drake et al 2003, Appendix, Item 7 |  |
| PDSS08 | Drake et al 2003, Appendix, Item 8 |  |
| Sleep Disturbances Scale for Children (SDSC)  - Bruni, O., Ottaviano, S., Guidetti, V., Romoli, M., Innocenzi, M., Cortesi, F., & Giannotti, F. (1996). The Sleep Disturbance Scale for Children (SDSC). Construction and validation of an instrument to evaluate sleep disturbances in childhood and adolescence. *J Sleep Res*, *5*(4), 251-261. <https://doi.org/10.1111/j.1365-2869.1996.00251.x>   NOTE: “The child” has been replaced with “your child” throughout. Gender neutral pronouns (and corresponding verb) were used. | | |
| QTAB Variable | Scale Item Source | Comment |
| pSDSC01 | Bruni et al 1996, Appendix A, Item 1 |  |
| pSDSC02 | Bruni et al 1996, Appendix A, Item 2 |  |
| pSDSC03 | Bruni et al 1996, Appendix A, Item 3 |  |
| pSDSC04 | Bruni et al 1996, Appendix A, Item 4 |  |
| pSDSC05 | Bruni et al 1996, Appendix A, Item 5 |  |
| pSDSC06 | Bruni et al 1996, Appendix A, Item 6 |  |
| pSDSC07 | Bruni et al 1996, Appendix A, Item 7 |  |
| pSDSC08 | Bruni et al 1996, Appendix A, Item 8 |  |
| pSDSC09 | Bruni et al 1996, Appendix A, Item 9 |  |
| pSDSC10 | Bruni et al 1996, Appendix A, Item 10 |  |
| pSDSC11 | Bruni et al 1996, Appendix A, Item 11 |  |
| pSDSC12 | Bruni et al 1996, Appendix A, Item 12 |  |
| pSDSC13 | Bruni et al 1996, Appendix A, Item 13 |  |
| pSDSC14 | Bruni et al 1996, Appendix A, Item 14 |  |
| pSDSC15 | Bruni et al 1996, Appendix A, Item 15 |  |
| pSDSC16 | Bruni et al 1996, Appendix A, Item 16 |  |
| pSDSC17 | Bruni et al 1996, Appendix A, Item 17 |  |
| pSDSC18 | Bruni et al 1996, Appendix A, Item 18 | “his/her” replaced with “their” |
| pSDSC19 | Bruni et al 1996, Appendix A, Item 19 |  |
| pSDSC20 | Bruni et al 1996, Appendix A, Item 20 | “him/her” replaced with “them” |
| pSDSC21 | Bruni et al 1996, Appendix A, Item 21 | “he/she” replaced with “they” |
| pSDSC22 | Bruni et al 1996, Appendix A, Item 22 |  |
| pSDSC23 | Bruni et al 1996, Appendix A, Item 23 |  |
| pSDSC24 | Bruni et al 1996, Appendix A, Item 24 |  |
| pSDSC25 | Bruni et al 1996, Appendix A, Item 25 | “somnolence” was replaced with “sleepiness” |
| pSDSC26 | Bruni et al 1996, Appendix A, Item 26 |  |
| Sleep behaviours across early childhood Adapted from Generation R sleep items reported in:   - Kocevska, D., Muetzel, R. L., Luik, A. I., Luijk, M. P., Jaddoe, V. W., Verhulst, F. C., White, T., & Tiemeier, H. (2017). The Developmental Course of Sleep Disturbances Across Childhood Relates to Brain Morphology at Age 7: The Generation R Study. *Sleep*, *40*(1). <https://doi.org/10.1093/sleep/zsw022>   Generation R items: Has trouble getting to sleep (QTAB item1); Sleeps less than most kids during day and/or night (QTAB item 2); Wakes up often at night (QTAB item 3); Doesn’t want to sleep alone (QTAB item 4); Resists going to bed at night (QTAB item 5); Parental presence required at bedtime (QTAB item 6) | | |
| QTAB Variable | Item | Comment |
| pGenR_2mth | Sleep behaviour sum score at age 2 months | Sum of variables  pGenR10 (Item 1 at age 2 months)  pGenR20 (Item 2 at age 2 months)  pGenR30 (Item 3 at age 2 months)  pGenR40 (Item 4 at age 2 months) |
| pGenR_18mth | Sleep behaviour sum score at age 18 months | Sum of variables  pGenR11 (Item 1 at age 18 months)  pGenR21 (Item 2 at age 18 months)  pGenR31 (Item 3 at age 18 months)  pGenR41 (Item 4 at age 18 months)  pGenR51 (Item 5 at age 18 months)  pGenR61 (Item 6 at age 18 months) |
| pGenR_2yrs | Sleep behaviour sum score at age 2 years | Sum of variables  pGenR12 (Item 1 at age 2 years)  pGenR22 (Item 2 at age 2 years)  pGenR32 (Item 3 at age 2 years)  pGenR42 (Item 4 at age 2 years)  pGenR52 (Item 5 at age 2 years)  pGenR62 (Item 6 at age 2 years) |
| pGenR_3yrs | Sleep behaviour sum score at age 3 years | Sum of variables  pGenR13 (Item 1 at age 3 years)  pGenR23 (Item 2 at age 3 years)  pGenR33 (Item 3 at age 3 years)  pGenR43 (Item 4 at age 3 years)  pGenR53 (Item 5 at age 3 years)  pGenR63 (Item 6 at age 3 years) |
| pGenR_6yrs | Sleep behaviour sum score at age 6 years | Sum of variables  pGenR16 (Item 1 at age 6 years)  pGenR26 (Item 2 at age 6 years)  pGenR36 (Item 3 at age 6 years)  pGenR46 (Item 4 at age 6 years)  pGenR56 (Item 5 at age 6 years)  pGenR66 (Item 6 at age 6 years) |
| pGenR_9yrs | Sleep behaviour sum score at age 9 years | Sum of variables  pGenR19 (Item 1 at age 9 years)  pGenR29 (Item 2 at age 9 years)  pGenR39 (Item 3 at age 9 years)  pGenR49 (Item 4 at age 9 years)  pGenR59 (Item 5 at age 9 years)  pGenR69 (Item 6 at age 9 years) |
| COVID-19 Pandemic Specific Assessments (subsample only) | | |
| Active and Passive Social Media Use (APSMU)  - Thorisdottir, I. E., Sigurvinsdottir, R., Asgeirsdottir, B. B., Allegrante, J. P., & Sigfusdottir, I. D. (2019). Active and Passive Social Media Use and Symptoms of Anxiety and Depressed Mood Among Icelandic Adolescents. *Cyberpsychol Behav Soc Netw*, *22*(8), 535-542. <https://doi.org/10.1089/cyber.2019.0079> - Frison, E., & Eggermont, S. (2015). Toward an Integrated and Differential Approach to the Relationships Between Loneliness, Different Types of Facebook Use, and Adolescents’ Depressed Mood. *Communication Research*, *47*(5), 701-728. <https://doi.org/10.1177/0093650215617506> | | |
| QTAB Variable | Item | Comment |
| APSMU01 | Thorisdottir et al 2019, Table 1, 1^st^ Item |  |
| APSMU02 | Thorisdottir et al 2019, Table 1, 2^nd^ Item |  |
| APSMU03 | Thorisdottir et al 2019, Table 1, 3^rd^ Item |  |
| APSMU04 | Thorisdottir et al 2019, Table 1, 4^th^ Item |  |
| APSMU05 | Thorisdottir et al 2019, Table 1, 5^th^ Item |  |
| APSMU06 | Thorisdottir et al 2019, Table 1, 6^th^ Item |  |
| UCLA Brief COVID-19 Screen for Child/Adolescent PTSD  - UCLA Brief COVID-19 Screen for Child/Adolescent PTSD. (2020). The Regents of the University of California. - <https://istss.org/getattachment/Clinical-Resources/Assessing-Trauma/UCLA-Posttraumatic-Stress-Disorder-Reaction-Index/UCLA-Brief-COVID-19-Screening-Form-English-4-13-20.pdf> (accessed 24^th^ November, 2022) | | |
| QTAB Variable | Item | Comment |
| PTSD01 | Online pdf, Page 2, Item 1 |  |
| PTSD02 | Online pdf, Page 2, Item 2 |  |
| PTSD03 | Online pdf, Page 2, Item 3 |  |
| PTSD04 | Online pdf, Page 2, Item 4 |  |
| PTSD05 | Online pdf, Page 2, Item 5 |  |
| PTSD06 | Online pdf, Page 2, Item 6 |  |
| PTSD07 | Online pdf, Page 2, Item 7 |  |
| PTSD08 | Online pdf, Page 2, Item 8 |  |
| PTSD09 | Online pdf, Page 2, Item 9 |  |
| PTSD010 | Online pdf, Page 2, Item 10 |  |
| PTSD011 | Online pdf, Page 2, Item 11 |  |
| Perceived Stress Scale (PSS)  - Cohen, S., Kamarck, T., & Mermelstein, R. (1983). A global measure of perceived stress. *J Health Soc Behav*, *24*(4), 385-396. <https://www.ncbi.nlm.nih.gov/pubmed/6668417> - Lee, B., & Jeong, H. I. (2019). Construct validity of the perceived stress scale (PSS-10) in a sample of early childhood teacher candidates. *Psychiatry and Clinical Psychopharmacology*, *29*(1), 76-82. <https://doi.org/10.1080/24750573.2019.1565693>   Note: the 10-item version of the PSS was used. | | |
| QTAB Variable | Scale Item | Comment |
| pPSS01 | Cohen et al 1983, Appendix A, Item 1  Lee et al 2019, Table 1, Item 1 |  |
| pPSS02 | Cohen et al 1983, Appendix A, Item 2  Lee et al 2019, Table 1, Item 2 |  |
| pPSS03 | Cohen et al 1983, Appendix A, Item 3  Lee et al 2019, Table 1, Item 3 |  |
| pPSS04 | Cohen et al 1983, Appendix A, Item 4  Lee et al 2019, Table 1, Item 4 | Lee et al 2019 version used |
| pPSS05 | Cohen et al 1983, Appendix A, Item 7  Lee et al 2019, Table 1, Item 5 | Lee et al 2019 version used |
| pPSS06 | Cohen et al 1983, Appendix A, Item 8  Lee et al 2019, Table 1, Item 6 |  |
| pPSS07 | Cohen et al 1983, Appendix A, Item 9  Lee et al 2019, Table 1, Item 7 |  |
| pPSS08 | Cohen et al 1983, Appendix A, Item 10  Lee et al 2019, Table 1, Item 8 |  |
| pPSS09 | Cohen et al 1983, Appendix A, Item 11  Lee et al 2019, Table 1, Item 9 | Lee et al 2019 version used |
| pPSS10 | Cohen et al 1983, Appendix A, Item 14  Lee et al 2019, Table 1, Item 10 |  |
| Brief Resilience Scale (BRS)  - Smith, B. W., Dalen, J., Wiggins, K., Tooley, E., Christopher, P., & Bernard, J. (2008). The brief resilience scale: assessing the ability to bounce back. *Int J Behav Med*, *15*(3), 194-200. <https://doi.org/10.1080/10705500802222972> | | |
| QTAB Variable | Scale Item | Comment |
| BRS01 (self-report)  pBRS01 (parent-report) | Smith et al 2008, Table 1, Item 1 |  |
| BRS02  pBRS02 | Smith et al 2008, Table 1, Item 2 |  |
| BRS03  pBRS03 | Smith et al 2008, Table 1, Item 3 |  |
| BRS04  pBRS04 | Smith et al 2008, Table 1, Item 4 |  |
| BRS05  pBRS05 | Smith et al 2008, Table 1, Item 5 |  |
| BRS06  pBRS06 | Smith et al 2008, Table 1, Item 6 |  |
